# Dopaminergic systems create reward seeking despite adverse consequences

**DOI:** 10.1038/s41586-023-06671-8

**Published:** 2023-10-25

**Authors:** Kristijan D. Jovanoski, Lucille Duquenoy, Jessica Mitchell, Ishaan Kapoor, Christoph D. Treiber, Vincent Croset, Georgia Dempsey, Sai Parepalli, Paola Cognigni, Nils Otto, Johannes Felsenberg, Scott Waddell

**Affiliations:** 1 Centre for Neural Circuits and Behaviour, University of Oxford, Oxford, UK; 2 Present Address: Friedrich Miescher Institute for Biomedical Research, Basel, Switzerland; 3 Present Address: Department of Biosciences, Durham University, Durham, UK; 4 Present Address: Northern Medical Physics and Clinical Engineering, Newcastle upon Tyne Hospitals NHS Trust, Newcastle upon Tyne, UK; 5 Present Address: Institute of Anatomy and Molecular Neurobiology, Westfälische Wilhelms-University, Münster, Germany

**Keywords:** Motivation, Reward

## Abstract

Resource-seeking behaviours are ordinarily constrained by physiological needs and threats of danger, and the loss of these controls is associated with pathological reward seeking^1^. Although dysfunction of the dopaminergic valuation system of the brain is known to contribute towards unconstrained reward seeking^2,3^, the underlying reasons for this behaviour are unclear. Here we describe dopaminergic neural mechanisms that produce reward seeking despite adverse consequences in *Drosophila melanogaster*. Odours paired with optogenetic activation of a defined subset of reward-encoding dopaminergic neurons become cues that starved flies seek while neglecting food and enduring electric shock punishment. Unconstrained seeking of reward is not observed after learning with sugar or synthetic engagement of other dopaminergic neuron populations. Antagonism between reward-encoding and punishment-encoding dopaminergic neurons accounts for the perseverance of reward seeking despite punishment, whereas synthetic engagement of the reward-encoding dopaminergic neurons also impairs the ordinary need-dependent dopaminergic valuation of available food. Connectome analyses reveal that the population of reward-encoding dopaminergic neurons receives highly heterogeneous input, consistent with parallel representation of diverse rewards, and recordings demonstrate state-specific gating and satiety-related signals. We propose that a similar dopaminergic valuation system dysfunction is likely to contribute to maladaptive seeking of rewards by mammals.

## Abstract (web summary)

In *Drosophila*, a subpopulation of reward-encoding dopaminergic neurons antagonizes punishment-encoding neurons and can override punishment or hunger cues in favour of reward-seeking behaviour.

## Main

Unconstrained reward-seeking behaviour in humans is typically associated with substance use disorders^3,4^. Rodents trained with electrical or optogenetic self-stimulation of their dopaminergic neurons (DANs) continue to self-administer stimulation even when punished, exhibiting behaviour similar to that following cocaine infusion^5–7^. Such studies demonstrate the usefulness of directed DAN activation as a model to understand acquisition of unconstrained reward-seeking behaviour^3^, without potentially confounding broad and non-specific pharmacological consequences of reward or drug consumption^8^. However, the heterogeneity of DANs in the mammalian ventral tegmental area^9,10^ and the challenges of recording from and targeting distinct subpopulations^11^^,^^12^ present major hurdles for the identification of the precise neural mechanisms underlying unconstrained reward-seeking behaviour.

The reduced numerical complexity of the *Drosophila* dopaminergic system^13^ enables the study of mechanisms of reward memory and seeking at cellular resolution. As in mammals, natural or artificial engagement of particular *Drosophila* DANs provides reward teaching signals that assign positive valence to sensory stimuli, forming appetitive memories for these cues^14–16^. Both flies and mice also possess aversively reinforcing DANs whose activation conveys negative valence^10,17–20^. In the adult fly, the net rewarding DAN population is approximately tenfold larger than the DAN population representing aversion^13^.

Functional analyses and input connectivity reveal extensive heterogeneity within the reward-encoding DANs^13,21^ that appears to allow parallel coding of different types of rewarding stimuli and events, such as the sweet taste and nutrient value of sugar^15,16,22,23^, water^24^, courtship (in males)^25^, absence of expected punishment^21,26^, safety^27^ and relative aversive value^28^. Moreover, combinations of aversive and rewarding DANs provide control over appropriate need-specific behavioural expression of reward-seeking memories^24,29,30^ or food-seeking behaviours^31^. We postulated that simultaneous engagement of multiple reward-specific signals might generate a ‘compound reward’ memory and produce reward seeking despite adverse consequences.

## Reward seeking despite punishment

**Fig. 1:**
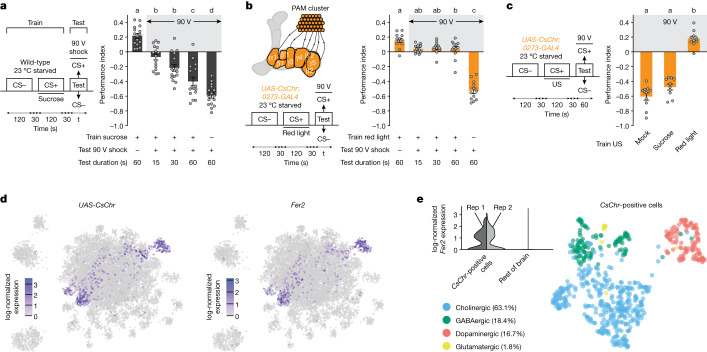
*Fer2*-expressing *0273* neurons drive reward seeking despite shock. **a**, Left, experimental protocol. Starved wild-type flies were trained to associate an odour (the CS+) with sucrose. t, test period. Right, learned CS+ approach can be competed with in a time-dependent manner by presenting the CS+ with 90 V shock (*n* = 16). Groups on the far left and far right show 60 s tests of sucrose-trained flies without electrified CS+ and 60 s shock avoidance of mock-trained flies, respectively. **b**, Top left, schematic of DANs labelled by *0273-GAL4* (other labelled neurons are not shown) that project from the PAM cluster to horizontal lobe mushroom body compartments. Bottom left, experimental protocol. Right, starved transgenic flies trained with CsChr activation of *0273* neurons do not show a time-dependent increase in CS+/90 V avoidance (*n* = 12). **c**, Left, experimental protocol. US, unconditioned stimulus. Right, starved flies trained with *0273*-neuron activation approach reward-predicting CS+ despite 90 V shock. Mock-trained and sucrose-trained flies exhibit shock avoidance (*n* = 10). Different letters above bars in **a**–**c** indicate groups that are significantly different from each other (*P* < 0.05; one-way ANOVA then Tukey’s honestly significant difference (HSD)). Data are mean ± s.e.m.; dots are individual data points that correspond to independent behavioural experiments. Exact statistical values and comparisons are presented in Supplementary Information. **d**, UMAP projections of scRNA-seq data show that neuron-driven *CsChr* expression (left) overlaps with *Fer2* expression (right). **e**, Top left, *CsChr*-positive cells express *Fer2* in both biological replicates (Rep 1 and Rep 2) whereas *Fer2* expression is almost absent in the rest of the brain. Right, *CsChr*-expressing cells co-express marker genes for cholinergic (63.1% of all cells), GABAergic (18.4%), dopaminergic (16.7%) or glutamatergic (1.8%) neurons.

A hallmark of unconstrained reward seeking is tolerance of adverse conditions such as electric shock while pursuing reward^3,32^. Associative olfactory learning with ethanol reward produces reward seeking despite shock in *Drosophila*^33^. We therefore tested whether electric shock punishment competed with approach towards an odour that was assigned a positive valence by recent olfactory learning with sucrose reward. Food-deprived wild-type flies were trained by presenting them with an odour alone (the conditioned stimulus minus (CS−)), followed by air and then another odour (the conditioned stimulus plus (CS+)) paired with dried sucrose (the unconditioned stimulus). Trained flies were then immediately tested in a T-maze for preference (for a duration of 15, 30 or 60 s, assuming shock avoidance increases with time) between CS+ odour presented with 90 V shocks (1.5 s duration every 5 s, standard conditions for a 60 s aversive olfactory training session; Methods) and CS− odour (Fig. 1a). Avoidance of electrified but sucrose-predicting odour progressively increased with testing duration. Wild-type flies therefore desist from seeking sucrose reward in the presence of 90 V shocks.

**Extended Data Fig. 1:**
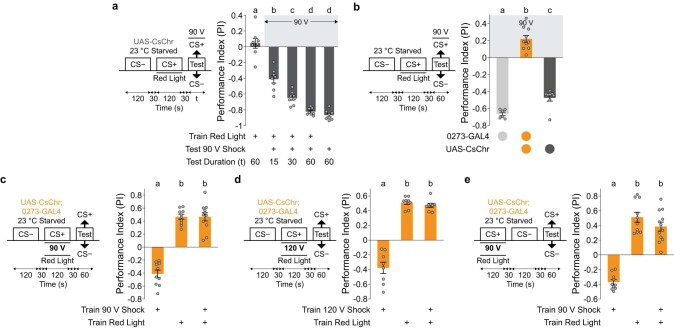
*0273* neurons drive reward seeking despite shock during training or testing. **a**, Left: Protocol. Right: Starved *UAS-CsChr* control flies show a time-dependent increase in CS+/90 V avoidance (*n* = 8). **b**, Left: Protocol. Right: Starved flies trained with *0273*-neuron activation approach the reward-predicting CS+ despite 90 V shock during testing compared with genetic controls (*n* = 6, 10, 8). **c**, Left: Protocol. Right: Starved flies trained with odour and *0273*-neuron stimulation display strong conditioned approach even when 90 V shocks are presented with the CS+ during training (*n* = 10, 10, 11). **d**, Left: Protocol. Right: Starved flies trained with odour and *0273*-neuron stimulation display strong conditioned approach after 120 V shocks are presented with the CS+ during training (*n* = 8). **e**, Left: Protocol. Right: Similar results are observed when the sequence of CS+ and CS− odours are reversed during 90 V training (*n* = 10, 10, 12). Different letters above bars indicate significantly different groups (*p* < 0.05; one-way ANOVA then Tukey’s HSD). All data mean ± SEM; dots are individual data points that correspond to independent behavioural experiments. Exact statistical values and comparisons in Supplementary Information.

Prevous work established that *0273-GAL4* labels around 130 largely reward-encoding DANs in the protocerebral anterior medial (PAM) cluster^16,23^. Artificial activation of the neurons labelled by *0273-GAL4* (hereafter termed *0273* neurons) reinforces robust olfactory memories^16,23^ and place memories^34^. We therefore used the red-light-sensitive cation channel CsChrimson (CsChr) to test whether flies would resist shock to seek the artificial reward of *0273*-neuron stimulation. Food-deprived *UAS-CsChr; 0273-GAL4* flies were trained by pairing an odour (CS+) with pulsed red light (optogenetic stimulation) instead of sucrose. As before, flies were then immediately tested for preference (for 15, 30 or 60 s) between the now-electrified CS+ odour and the non-electrified CS− odour (Fig. 1b). Surprisingly, around 50% of the trained *UAS-CsChr; 0273-GAL4* flies (a zero performance index; Fig. 1b) consistently approached the electrified reward-predicting odour irrespective of testing duration, whereas genetic controls robustly avoided shock (Extended Data Fig. 1a,b). Thus, flies persist in seeking *0273*-neuron reward despite ongoing punishment.

We next compared *0273*-neuron-driven shock-resistant odour approach for a test duration of 60 s to that of mock-trained (no unconditioned stimulus) and sucrose-trained flies. Optogenetically trained *UAS-CsChr; 0273-GAL4* flies showed a preference for the electrified reward-predicting CS+ odour, whereas mock-trained and sucrose-trained flies avoided it (Fig. 1c). Therefore, training *UAS-CsChr; 0273-GAL4* flies with a natural reward such as sucrose does not recapitulate synthetic *0273*-neuron-driven shock-resistant reward seeking.

Tolerance of 90 V shocks to seek *0273*-neuron-reinforced reward suggests that the predicted value of *0273*-neuron reward is high. We therefore tested whether the *0273*-neuron reward teaching signal could be countered by simultaneously presenting optogenetic activation and shocks during training. Flies trained with odour and *0273*-neuron stimulation displayed strong conditioned approach even when 90 or 120 V shocks were presented simultaneously during training (Extended Data Fig. 1c,d), or when the sequence of CS+ and CS− odours was reversed during 90 V training (Extended Data Fig. 1e). Thus, *0273* neurons reinforce reward seeking that is resistant to simultaneous or subsequent shock punishment.

## *0273-GAL4* labels mixed neuronal types

*0273-GAL4* flies carry a PBac{IT.GAL4} element inserted into the *Fer2* gene, which encodes the 48-related 2 (Fer2) basic helix-loop-helix transcription factor^35^. *Fer2* is expressed in and required for the development of DANs with cell bodies in the PAM and protocerebral anterior lateral (PAL) clusters^36^. However, *0273-GAL4* also drives expression in other neurons in the brain, including the ventral lateral neurons of the circadian clock^37^. We therefore used 10x Genomics Chromium single-cell RNA sequencing (scRNA-seq) to characterize the cell types labelled by *0273*-driven CsChr.

From two independent biological replicates comprising 32 fly central brains in total, we obtained gene-expression signatures for 11,502 cells with an average of 5,673 transcripts detected per cell. Barcoded sequencing reads were aligned to the *Drosophila* reference genome and to the *CsChr* transgene. Plotting *CsChr* expression onto a uniform manifold approximation and projection (UMAP) reduction of the data revealed a strong correlation with *Fer2* expression (Fig. 1d,e), demonstrating that *0273-GAL4* faithfully recapitulates *Fer2* expression. Expression of the DAN marker genes *vesicular monoamine transporter* (*Vmat*) and *dopamine transporter* (*DAT*) revealed that around 16.7% of *CsChr*-expressing cells were DANs (Fig. 1e). Other *CsChr*-positive but *Vmat*-negative and *DAT*-negative cells expressed synthesis and packaging markers for the fast-acting neurotransmitters acetylcholine (approximately 63.1% of cells), γ-aminobutyric acid (GABA) (approximately 18.4%) and glutamate (approximately 1.8%).

**Extended Data Fig. 2:**
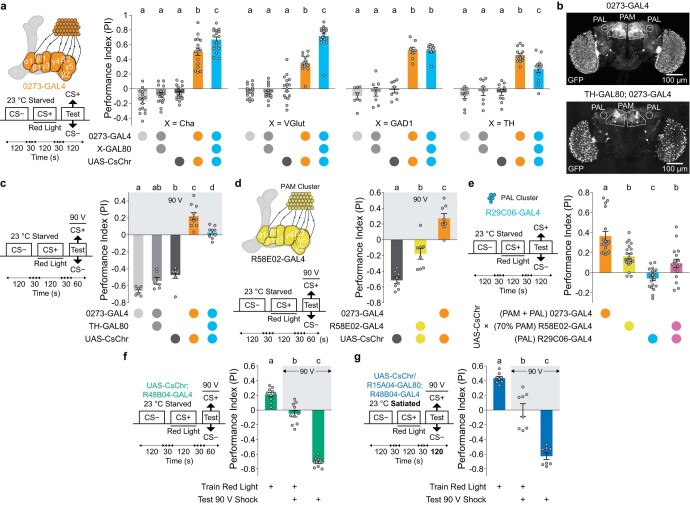
Identification of DANs that drive reward seeking despite shock. **a**, Left: Schematic and protocol. Right: *GAL80* co-expression with *0273-GAL4* reveals that removing cholinergic expression with *Cha-GAL80* (*n* = 16) or glutamatergic expression with *VGlut-GAL80* (*n* = 16) significantly enhances 0273-reinforced memory, removing GABAergic expression with *GAD1-GAL80* (*n* = 8) has no effect, and removing dopaminergic expression with *TH-GAL80* (*n* = 10, 8, 10, 14, 12) reduces memory. **b**, Combining *TH-GAL80* with *0273-GAL4* visibly reduces GFP expression in DANs in the PAM and PAL clusters (dashed shapes). Representative images from one of two brains for each genotype shown. **c**, Left: Protocol. Right: *TH-GAL80* impairs 0273-driven shock-resistant reward seeking (*n* = 6, 6, 8, 10, 10). **d**, Left: Protocol and schematic of *R58E02-GAL4*, which labels ~70% of PAM DANs. Right: *R58E02*-reinforced memory does not override avoidance of the shock-paired CS+ as effectively as *0273*-reinforced memory (*n* = 8). **e**, Left: Protocol and schematic of PAL cluster DANs labelled by *R29C06-GAL4* (other labelled neurons not shown). Right: *R58E02*-labelled PAM DAN and *R29C06*-labelled PAL DAN coactivation does not reproduce memory performance after *0273* activation (*n* = 18, 20, 20, 16). **f**, Left: Protocol. Right: Starved flies trained with activation of *R48B04* neurons seek reward despite simultaneous 90 V shock (*n* = 10). **g**, Left: Protocol. Right: Satiated flies trained with activation of β′2&γ4 DANs seek reward for 120 s despite 90 V shock (*n* = 8). Different letters above bars indicate significantly different groups (*p* < 0.05; one-way ANOVA then Tukey’s HSD). All data mean ± SEM; dots are individual data points that correspond to independent behavioural experiments. Exact statistical values and comparisons in Supplementary Information.

Previous work implicated *0273-GAL4*-expressing DANs in reinforcing olfactory memory^16,23^ but implicated both DANs and cholinergic neurons in conditioned place preference^34^. We therefore tested whether different types of *0273*-labelled neuron contributed towards artificially implanted appetitive olfactory memory (tested for odour preference without shock, for a standard 120 s; Methods). We prevented GAL4-mediated expression using cell-type-specific co-expression of GAL80, which represses GAL4-mediated transcription. Removing GAL4-mediated expression from cholinergic or glutamatergic neurons, but not from GABAergic neurons, increased *0273*-neuron-induced appetitive memory (Extended Data Fig. 2a). Performance enhancement suggests that *0273*-neuron-mediated reward is limited by concurrent activation of *0273*-labelled cholinergic or glutamatergic neurons.

By contrast, removing some DAN expression with a *pale* (tyrosine hydroxylase (*TH*)) promoter-fragment-driven GAL80 (*TH-GAL80*) impaired *0273*-neuron-implanted appetitive memory (Extended Data Fig. 2a). Consistent with a previous description that *TH*-*GAL4* labels PPL1 DANs (not labelled by *0273-GAL4*^16^), 13 PAM DANs per hemisphere and PAL DANs^17^, confocal imaging revealed that *TH-GAL80* reduced *0273-GAL4* labelling in PAM and PAL DANs (Extended Data Fig. 2b). Nonetheless, we note that *GAL80* transgenes do not always faithfully reproduce the expression patterns of *GAL4* transgenes driven by the same promoter fragment^23,38^. We also found that *TH-GAL80* reduced *0273*-neuron-driven shock-resistant reward seeking (Extended Data Fig. 2c). Therefore, DANs that are targeted by *TH-GAL80* are required for *0273-*driven reward seeking.

*R58E02-GAL4* labels around 90 rewarding PAM DANs that largely overlap with the approximately 130 DANs labelled by *0273-GAL4*^15,23^. However, we observed that flies trained with activation of *R58E02-GAL4* neurons only partially avoided the electrified CS+ odour during testing (Extended Data Fig. 2d), suggesting that *R58E02-GAL4* labels only some of the PAM DANs required for *0273*-driven reward seeking. Moreover, red-light activation of PAL DANs together with odour did not produce appetitive memory or augment *R58E02-GAL4* PAM-DAN-implanted memory (Extended Data Fig. 2e).

## Specific DANs account for reward seeking

**Fig. 2:**
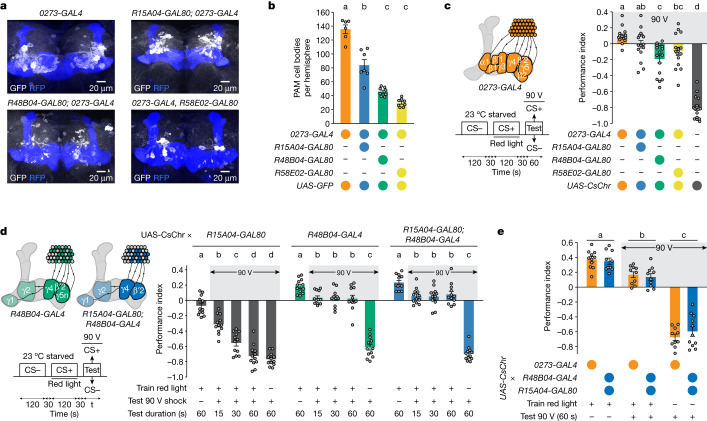
Specific PAM DANs recapitulate *0273*-neuron-mediated reward seeking. **a**, Representative GFP expression in DANs driven by *0273*-*GAL4* combined with different *GAL80* transgenes: *R15A04-GAL80*, *R48B04-GAL80* or *R58E02-GAL80*. Mushroom body is co-labelled with RFP for reference. Three brains were examined for *0273-GAL4*, four brains were examined for the other genotypes. **b**, *R58E02-GAL80* produces the greatest reduction in number of PAM somata per hemisphere labelled by *0273-GAL4*, followed by *R48B04-GAL80* then *R15A04*-*GAL80* (left to right: *n* = 6, 8, 8 and 8). **c**, Left, schematic and experimental protocol. Right, *R48B04-GAL80* produces the greatest shock-induced reversal of reward seeking driven by *0273* neurons, followed by *R58E02-GAL80*. *R15A04-GAL80* has no significant effect (*n* = 16). **d**, Left, schematics and experimental protocol. Bottom, starved flies trained with *R48B04-GAL4* (with or without *R15A04-GAL80*) neuron activation do not show the time-dependent increase in CS+/90 V avoidance observed in *R15A04*-*GAL80* controls (*n* = 12). Different letters above bars in **b**–**d** indicate groups that are significantly different from each other (*P* < 0.05; one-way ANOVA then Tukey’s HSD; comparisons in **d** only within genotypes). **e**, Preference for CS+/90 V is similar for flies harbouring memory implanted by activation of *0273* neurons or β′2&γ4 DANs (left to right: *n* = 11, 10, 10, 10, 10 and 11; protocol as in **c**). Different letters above bars indicate treatments that are significantly different (*P* < 0.05; two-way ANOVA then Tukey’s HSD; main effect of treatment: *F*(2,56) = 262.1, *P* < 0.0001). Data are mean ± s.e.m.; dots are individual data points that correspond to individual hemispheres (**b**) or independent behavioural experiments (**c**–**e**). Exact statistical values and comparisons are presented in Supplementary Information.

We used other *GAL80* transgenes to identify PAM DANs involved in shock-resistant reward seeking. We first assessed GAL80-mediated suppression of *0273-GAL4* by counting PAM cells that remain labelled in intersections with *UAS-mCD8::GFP* (Fig. 2a). *R15A04-GAL80* reduced the number of *0273-GAL4*-labelled PAM DANs from around 130 to approximately 84 cells, whereas approximately 45 PAM DANs remained with *R48B04-GAL80*, and approximately 30 remained with *R58E02-GAL80* (Fig. 2b). We next constructed *UAS-CsChr; 0273-GAL4* flies that also carried *R15A04-GAL80*, *R48B04-GAL80* or *R58E02-GAL80* and trained them by pairing CS+ odour with red light to stimulate neurons with functional GAL4 before testing them for shock-resistant reward approach (Fig. 2c). The *0273*-neuron-driven conditioned approach was attenuated by removing CsChr expression in PAM DANs with all of the *GAL80* transgenes and most strongly by *R48B04-GAL80*. The behaviour therefore focused our attention on the role of *R48B04*-labelled PAM DANs in the development of shock-resistant reward seeking.

*R48B04-GAL4* drives expression in approximately 55 PAM DANs^23,24^ (and approximately 12 *TH*-negative neurons)—comprising most DANs that innervate the β′2 and γ4 compartments in the horizontal mushroom body lobe—and a subset of DANs innervating γ5 that were previously designated ‘γ5 narrow’^23^ (γ5n). *R48B04* DANs innervating β′2 or γ4 compartments are necessary for acquiring short-term olfactory associations with sugar^22,23^ and water^24^. β′2 DANs also control feeding rate and satiation^39^, and regulate the expression of appetitive alcohol-associated memory^40^, whereas artificial PAM DAN activation changes olfactory responses in the γ4 compartment^41,42^. *R15A04-GAL80* removes GAL4-mediated expression in γ5n DANs in *R48B04-GAL4* flies but leaves GAL4 activity in β′2 and γ4 DANs intact^24^. We also noted that *R48B04* neurons are likely to overlap with some PAM DANs labelled by *TH-GAL4* (and therefore possibly also *TH*-*GAL80*) that project to the β′2 and γ5 compartments^17^.

We next used *R48B04-GAL4* with *R15A04-GAL80* to test the role of β′2, γ4 and γ5 DANs in the development of shock-resistant reward seeking. Food-deprived flies carrying *R15A04-GAL80* or *R48B04-GAL4* or both (that is, with and without expression in γ5n DANs) were trained with odour paired with CsChr-mediated neuron activation (Fig. 2d). As previously observed for *UAS-CsChr; 0273-GAL4* flies (Fig. 1b), *UAS-CsChr; R48B04-GAL4* flies (with or without *R15A04-GAL80*) endured shock for 15, 30 or 60 s to seek the expected reward (Fig. 2d and Extended Data Fig. 2f). By contrast, control flies expressing only *R15A04-GAL80* progressively avoided the electrified CS+ (Fig. 2d), similar to sucrose-trained wild-type flies (Fig. 1a). Moreover, flies with CsChr-mediated activation of *R15A04-GAL80; R48B04-GAL4* neurons (hereafter termed β′2&γ4 DANs) persisted in seeking the electrified CS+ for 120 s and when food-satiated (Extended Data Fig. 2g). Direct comparison of reward seeking of flies trained with CsChr-mediated activation of *0273* neurons or only β′2&γ4 DANs revealed their conditioned CS+ approach, CS+ approach in the presence of 90 V and mock-trained shock avoidance to be equivalent between genotypes (Fig. 2e). Artificial shock-resistant reward-seeking memory can therefore be implanted in a state-independent manner by β′2&γ4 DANs.

**Extended Data Fig. 3:**
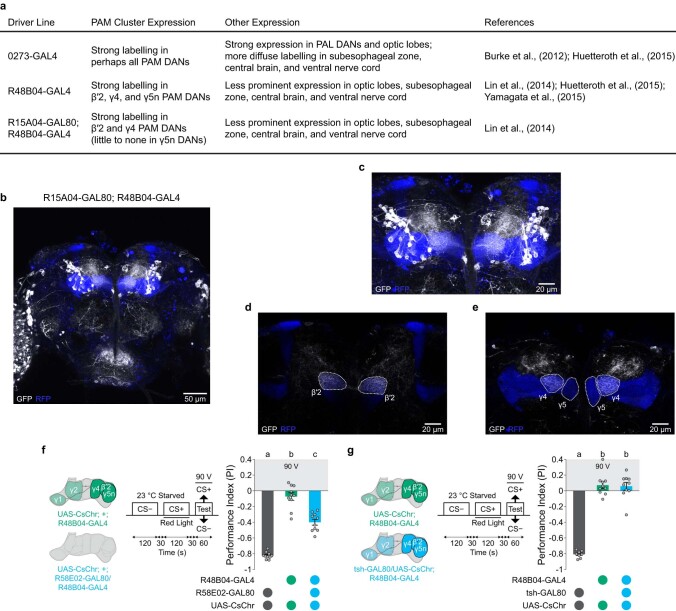
Expression patterns of PAM DAN driver lines that are required for shock-resistant reward seeking. **a**, Table summarizing all PAM cluster expression and other expression for each driver line whose artificial activation reinforces shock-resistant reward seeking. References containing images of expression patterns are listed. **b**, Representative GFP expression (white) driven by *R48B04-GAL4* combined with *R15A04-GAL80*, and the mushroom body (blue) co-labelled with RFP for reference. **c**, Magnified mushroom body GFP expression driven by *R15A04-GAL80; R48B04-GAL4*. **d**, *R15A04-GAL80; R48B04-GAL4* drives GFP expression in β′2 DANs (dashed shapes). **e**, *R15A04-GAL80; R48B04-GAL4* also drives GFP expression in γ4 DANs but not γ5 DANs (dashed shapes). The representative images in **b**, **c**, **d**, **e** are reproduced from source confocal data of one of two brains from ref. ^24^. **f**, Left: Schematics and protocol. Right: *UAS-CsChr; R48B04-GAL4* flies artificially trained with red light exhibit shock-resistant reward seeking that is impaired by *R58E02-GAL80* coexpression (*n* = 10). **g**, Left: Schematics and protocol. Right: *tsh-GAL80* coexpression did not impair shock-resistant reward seeking driven by *UAS-CsChr; R48B04-GAL4* (*n* = 10, 10, 8). Different letters above bars indicate significantly different groups (*p* < 0.05; one-way ANOVA then Tukey’s HSD). All data mean ± SEM; dots are individual data points that correspond to independent behavioural experiments. Exact statistical values and comparisons in Supplementary Information.

Since *R48B04-GAL4*—with or without *R15A04-GAL80—*also drives expression in non-PAM cells elsewhere in the nervous system (Extended Data Fig. 3a–e), we used *GAL80* transgenes to examine the role of these other cells in shock-resistant reward seeking in *UAS-CsChr; R48B04-GAL4* flies after optogenetic training. *R58E02-GAL80*, which represses *R48B04* labelling in the PAM DANs^22^, significantly impaired *R48B04*-driven shock-resistant reward seeking (Extended Data Fig. 3f), whereas *teashirt* (*tsh)*-*GAL80*, which represses *R48B04*-driven expression in the ventral nerve cord^43^, had no effect (Extended Data Fig. 3g). We therefore conclude that shock-resistant reward seeking requires *R48B04*-driven expression in PAM DANs, although we cannot completely exclude possible contributions from other neurons in the brain.

**Extended Data Fig. 4:**
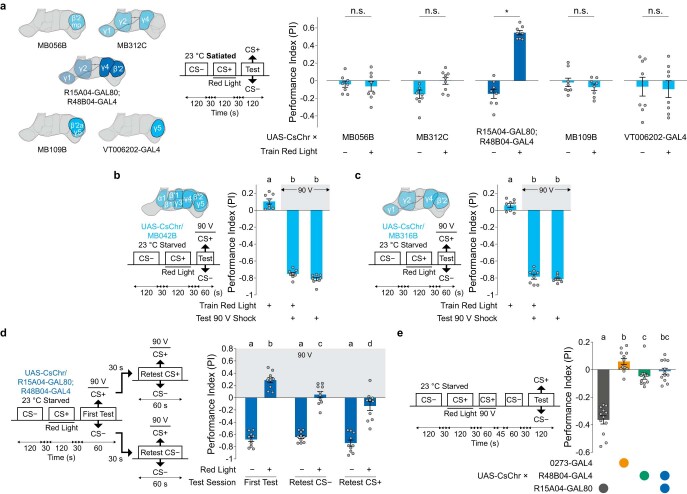
Flies trained with coactivation of sufficient β′2 and γ4 DANs seek reward even after experiencing the CS+ odour with shock. **a**, Left: Schematics and protocol. Right: Only training with activation of both β′2 and γ4 DANs paired with an odour leads to substantial conditioned approach (*n* = 8, *MB312C* *n* = 9). Breaks in x-axis demarcate separate experiments. Asterisks indicate significantly different groups (*p* < 0.05; two-sided unpaired t-test for each genotype with Holm-Šidák’s correction; n.s. = not significant). **b**, Left: Schematic and protocol. Right: Red-light-trained *MB042B* flies expressing *UAS-CsChr* exhibit only minor conditioned approach that is not shock-resistant (*n* = 8). **c**, Left: Schematic and protocol. Right: Red-light-trained *MB316B* flies expressing *UAS-CsChr* similarly exhibit minor conditioned approach that is not shock-resistant (*n* = 8). Different letters above bars in **b**, **c** indicate significantly different groups (*p* < 0.05; one-way ANOVA then Tukey’s HSD). **d**, Left: Consecutive testing protocol. Right: Flies with β′2&γ4 DAN-implanted memories subjected to consecutive testing in the presence of shock continue to approach the electrified CS+ odour irrespective of their first test choice (*n* = 10). Different letters above bars indicate significantly different groups (*p* < 0.05; two-way ANOVA then multiple comparisons with Šidák’s correction). Note that not all flies in the Retest CS− groups have necessarily experienced the CS+ during the first test (and vice versa for the Retest CS+ groups). **e**, Left: Consecutive training protocol. Right: Flies with β′2&γ4 DAN implanted memory and then trained to associate the CS+ with shock continue to approach the reward-predicting CS+ compared with control flies (*n* = 12). Different letters above bars indicate significantly different groups (*p* < 0.05; one-way ANOVA then Tukey’s HSD). All data mean ± SEM; dots are individual data points that correspond to independent behavioural experiments. Exact statistical values and comparisons in Supplementary Information.

We next attempted to reconstitute expression in β′2&γ4 DANs using more restricted split-GAL4 lines. However, each available line labels only a fraction of the DANs that innervate the β′2 and γ4 compartments (for example, *MB312C* labels 13 out of 31 known γ4 DANs^13,44^). Optogenetic training of satiated flies expressing CsChr driven by the split-GAL4 lines *MB056B* (which labels PAM-β′2m and PAM-β′2p DANs), *MB109B* (PAM-β′2 and PAM-γ5 DANs), *MB312C* (PAM-γ4 DANs) or *VT006202-GAL4* (PAM-γ5 DANs) did not produce detectable reward-seeking memory similar to that in *R15A04-GAL80; R48B04-GAL4* (PAM-β′2&γ4) trained flies (Extended Data Fig. 4a). In addition, *MB042B* and *MB316B* split-GAL4 lines, which both drive sparse expression in multiple PAM DAN subtypes—including some β′2 and γ4 DANs^44,45^—produced minor optogenetically induced memory that was not shock-resistant (Extended Data Fig. 4b,c). We therefore propose that β′2 and γ4 DANs must be activated together in sufficient numbers to drive shock-resistant reward seeking.

Finally, we investigated the robustness of β′2&γ4 DAN-implanted reward-seeking memories. We first subjected flies with β′2&γ4 DAN-implanted memories to consecutive testing (Extended Data Fig. 4d). Flies were optogenetically trained then tested for approach to CS+ odour with 90 V shock. They were then separated depending on their choice of entering either the electrified CS+ or non-electrified CS− T-maze arm during the first test and retested. About 50% of flies continued to approach the electrified CS+ odour irrespective of first test choice, although there was a modest decrease in CS+ approach compared with the first test (Extended Data Fig. 4d). We next asked whether β′2&γ4 DAN-implanted odour approach memory could be nullified with consecutive training in which the odour initially paired with red light was subsequently paired with shock. Flies with β′2&γ4 DAN-implanted memory continued to approach the reward-predicting odour (Extended Data Fig. 4e). Many flies trained with β′2&γ4 DAN stimulation therefore continue to pursue the reward-predicting CS+ even after experiencing the same odour with punishment.

## Reward DANs antagonize aversive DANs

In mammals, absence of expected reward leads to a decrease in DAN firing^14^, and acute inhibition of putatively reward-coding DANs can reinforce learned avoidance^46,47^. *Drosophila* DANs innervating the γ3 mushroom body compartment have been shown to reinforce aversive learning when transiently activated and to reinforce appetitive learning when inactivated^48^, although there may be two PAM-γ3 DAN subpopulations of opposing valence^13^. Since activation of *0273* neurons or β′2&γ4 DANs reinforced shock-resistant reward seeking (Figs. 1b and 2f), we investigated whether their inhibition could assign aversive value.

**Fig. 3:**
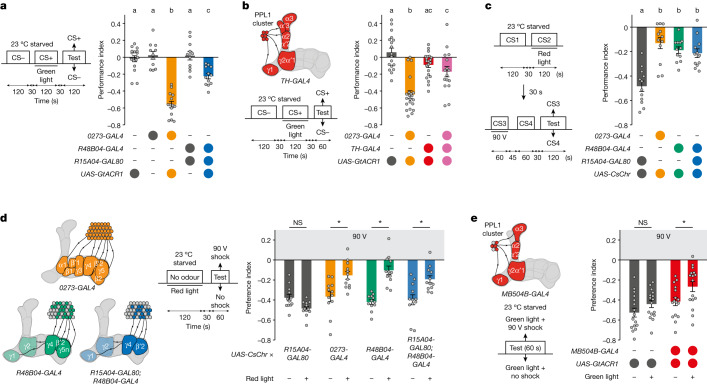
Reward DANs antagonize aversive DAN function. **a**, Left, schematics and protocol. Right, optogenetic silencing of *0273* neurons implants aversive memory for CS+ odour. Silencing β′2&γ4 DANs (*R15A04-GAL80; R48B04-GAL4*) forms aversive memory with less strength (left to right: *n* = 16, 11, 14, 11 and 12). **b**, Left, protocol and schematic of DANs labelled by *TH-GAL4* (other labelled neurons not shown) that project from PPL1 to vertical lobe mushroom body compartments. Right, optogenetic silencing of *TH-GAL4* DANs alone has no effect, whereas silencing both *0273* neurons and *TH-GAL4* neurons largely abrogates aversive memory implanted with *0273*-neuron silencing (left to right: *n* = 18, 21, 21, 16). **c**, Left, experimental protocol. Right, flies trained with artificial DAN activation do not learn a subsequent shock-paired CS+ as effectively as *R15A04-GAL80* controls (*n* = 12). Different letters above bars in **a**–**c** indicate groups that are significantly different from each other (*P* < 0.05; one-way ANOVA then Tukey’s HSD). **d**, Left, schematics and experimental protocol. Right, flies that experience optogenetic activation in an odourless tube show less subsequent shock avoidance than no-light controls of the same genotype (*n* = 12). **e**, Left, schematic and protocol. Right, flies with silenced PPL1 DANs exhibit less shock avoidance than controls (*n* = 16). **d**,**e**, *P < 0.05; two-way ANOVA then multiple comparisons with Šidák’s correction. NS, not significant. Data are mean ± s.e.m.; dots are individual data points that correspond to independent behavioural experiments. Exact statistical values and comparisons are presented in Supplementary Information.

Food-deprived flies expressing the green-light-sensitive chloride channel GtACR1 in *0273* neurons or in β′2&γ4 DANs were trained by presenting an odour alone (CS−), and then a second odour paired with continuous green light (optogenetic inhibition) (CS+). When tested immediately for odour preference, both genotypes of flies exhibited learned avoidance of CS+ odour (Fig. 3a), whereas controls showed no preference. Therefore, inhibition of *0273* neurons or β′2&γ4 DANs generates an aversive teaching signal.

Teaching signals for aversive stimuli such as electric shock are conveyed to the mushroom body by DANs in the protocerebral posterior lateral 1 (PPL1) cluster^17^. In addition, aversive and reward DANs often function antagonistically to guide appropriate memory-directed behaviour in *Drosophila*^26,27,29,30,49^. We therefore tested whether transient inhibition of aversive PPL1 DANs might interfere with learned avoidance generated by inhibiting reward DANs. Flies were trained by pairing odour with inhibition of *0273-GAL4* neurons or PPL1 DANs (labelled with *TH-GAL4*) or both during CS+ presentation (Fig. 3b). Notably, inhibiting both *0273* neurons and *TH* neurons during odour exposure abolished the learned CS+ aversion observed when *0273* neurons alone were inhibited. We did not observe reward learning after inhibiting *TH* neurons alone (Fig. 3b), suggesting that aversive DANs do not exert a mutual functional antagonism over the larger population of reward DANs. Together, these results suggest that learned avoidance following *0273*-neuron inhibition requires output from aversive PPL1 DANs.

The opposite valence of memories formed by activation or inhibition of *0273* neurons and β′2&γ4 DANs (Figs. 2e and 3a) and the evidence that forming aversive memories with *0273*-neuron inhibition required aversive PPL1 DAN output (Fig. 3b) led us to hypothesize that activation of *0273* neurons or β′2&γ4 DANs might indirectly inhibit PPL1 DAN function. Since PPL1 DANs are required for aversive olfactory shock learning^17^, we further reasoned that persistent shock-resistant reward seeking (Fig. 1b and 2d) could result from such an interaction with PPL1 DANs. We tested this model with a consecutive training paradigm. Food-deprived flies were trained with odour paired with CsChr-mediated activation of *0273* neurons, *R48B04* DANs or β′2&γ4 DANs, then immediately with aversive conditioning using a different odour pair, with one odour of the pair being combined with shock (Fig. 3c). Compared with *R15A04-GAL80* control flies, aversive memory was impaired in all groups that were previously trained with neuronal activation. Implanting memory with activation of *0273* neurons, *R48B04* DANs or β′2&γ4 DANs therefore compromises subsequent aversive learning reinforced by PPL1 DANs.

**Extended Data Fig. 5:**
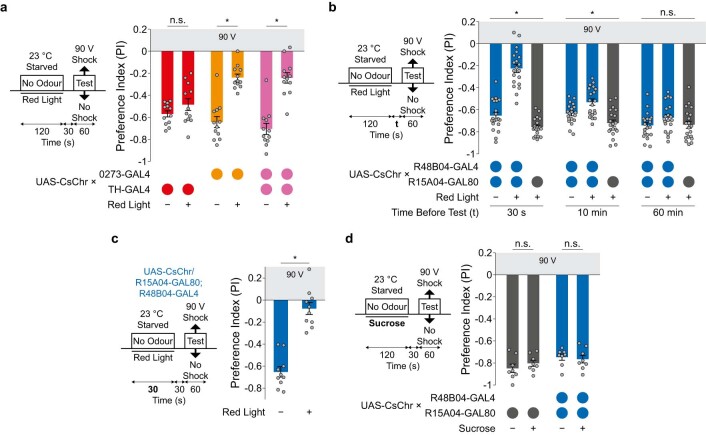
Reward DAN activation impedes subsequent shock avoidance. **a**, Left: Protocol. Right: Artificial activation of *TH* neurons does not affect subsequent naïve shock avoidance, nor does *TH* neuron coactivation with *0273* neurons restore shock avoidance performance (*n* = 12; * *p* < 0.05; two-way ANOVA then multiple comparisons with Šidák’s correction; n.s. = not significant). **b**, Left: Protocol with variable time *t* between red light activation and shock avoidance testing. Right: Naïve shock avoidance remains impaired compared with genetic and protocol controls 10 min after β′2&γ4 DAN activation but returns to normal levels by 60 min (*n* = 20; * *p* < 0.05; two-way ANOVA then Tukey’s HSD). **c**, Left: Protocol. Right: Naïve shock avoidance is impaired after β′2&γ4 DANs are activated for just 30 s (*n* = 10; * *p* < 0.05; two-sided unpaired t-test). **d**, Left: Protocol. Right: Sucrose presentation to starved flies for 120 s does not affect subsequent naïve shock avoidance in *UAS-CsChr/R15A04-GAL80; R48B04-GAL4* flies or *UAS-CsChr/R15A04-GAL80* flies (*n* = 8; *p* > 0.05; two-way ANOVA; main effect of treatment: *F*(1,28) = 104.9, *p* = 0.66). All data mean ± SEM; dots are individual data points that correspond to independent behavioural experiments. Exact statistical values and comparisons in Supplementary Information.

As aversive learning requires sensory processing of shock, we next tested whether artificially activating *0273* neurons, *R48B04* DANs or β′2&γ4 DANs without odour pairing might impede naive shock avoidance. Flies were exposed to red light for 120 s to activate *0273* neurons, *R48B04* DANs or β′2&γ4 DANs, then immediately tested for avoidance of 90 V shock without odour present (Fig. 3d). Prior activation of any of these groups of neurons impaired naive shock avoidance compared with *R15A04-GAL80* control flies (Fig. 3d). By contrast, prior activation of *TH* neurons did not affect subsequent naive shock avoidance, and *TH* neuron coactivation with *0273* neurons did not restore shock-avoidance performance (Extended Data Fig. 5a). Naive shock avoidance remained impaired 10 min after β′2&γ4 DAN activation but returned to normal levels by 1 h (Extended Data Fig. 5b), demonstrating that the inhibitory effect is transient. In addition, shock avoidance was also impaired following a shorter 30 s β′2&γ4 DAN activation (Extended Data Fig. 5c), but not by a 120 s presentation of sucrose to starved flies (Extended Data Fig. 5d). Together, these results suggest that independent of olfactory learning, transient simultaneous engagement of multiple classes of reward DANs can antagonize the function of aversive DANs. Moreover, natural rewards such as sucrose do not recapitulate this phenomenon.

We further tested whether optogenetic inhibition of PPL1 DANs (using *MB504B*-driven *UAS-GtACR1*) altered shock avoidance (Fig. 3e). Flies with inhibited PPL1 DANs revealed a significant impairment in naive shock avoidance compared with controls. This impairment of shock avoidance with acute aversive DAN inhibition mirrors that observed after reward DAN activation. Our data therefore suggest that persistent reward seeking despite shock arises from a dual process of the high expected reward value (or incentive value) of the artificially reinforced odour and the simultaneous impairment of neural processing of aversion.

## Reward DAN activity overrides need

**Fig. 4:**
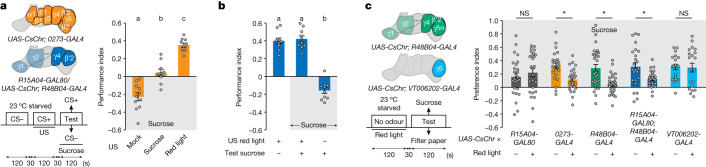
Reward DAN activity reduces subsequent need seeking. **a**, Left, schematics and experimental protocol. Right, starved flies trained with *0273*-neuron activation (orange) disregard sucrose to seek the CS+ odour predicting artificial reward, whereas flies trained with sucrose exhibit no preference, and mock-trained flies prefer sucrose (left to right: *n* = 11, 10 and 10). **b**, Starved flies trained with activation of β′2&γ4 DANs also disregard sucrose to seek artificial reward (*n* = 10, unconditioned stimulus is the red light protocol from **a** and genotype corresponds to the schematic with blue regions in **a**). Different letters above bars in **a**,**b** indicate groups that are significantly different from each other (*P* < 0.05; one-way ANOVA then Tukey’s HSD). **c**, Left, schematics and protocol. Right, activation of *0273* neurons, *R48B04* DANs, or β′2&γ4 DANs in an odourless tube decreases subsequent sucrose approach in starved flies (left to right: *n* = 26, 26, 26, 26, 28, 28, 24, 24, 20 and 20). Two-way ANOVA then multiple comparisons with Šidák’s correction. Data are mean ± s.e.m.; dots are individual data points that correspond to independent behavioural experiments. Exact statistical values and comparisons are presented in Supplementary Information.

**Extended Data Fig. 6:**
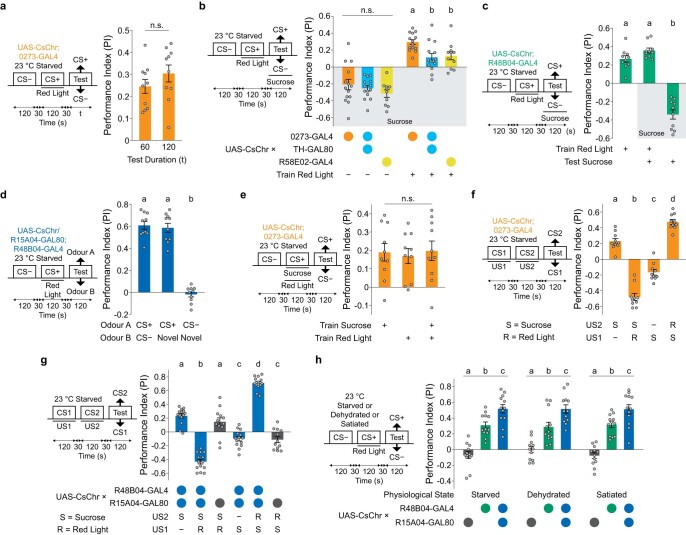
*0273* neurons and β′2&γ4 DANs drive reward seeking over sucrose. **a**, Left: Protocol. Right: Starved flies trained with odours and optogenetic activation of *0273* neurons exhibit similar reward approach for testing periods of 60 s or 120 s (*n* = 10; *p* > 0.05; two-sided unpaired t-test; n.s. = not significant). **b**, Left: Protocol. Right: Starved *UAS-CsChr; R58E02-GAL4* flies or *UAS-CsChr/TH-GAL80; 0273-GAL4* flies show reduced preference for the reward-predicting CS+ over the sucrose-laden CS− (*n* = 14, 14, 10, 14, 14, 11). **c**, Left: Protocol (same as Fig. 4b). Right: Starved flies trained with optogenetic activation of *R48B04* DANs disregard sucrose to seek artificial reward (*n* = 10). **d**, Left: Protocol. Right: Optogenetic training does not form an avoidance memory for the CS− odour (*n* = 10). **e**, Left: Protocol. Right: Optogenetic training does not potentiate simultaneous training with sucrose (*n* = 10; *p* > 0.05; one-way ANOVA). **f**, Left: Protocol. Right: Flies trained with one odour paired with *0273* activation and another odour paired with sucrose, prefer the previously red-light-paired odour at testing, irrespective of the training presentation sequence (*n* = 10). **g**, Left: Protocol. Right: Flies similarly trained with β′2&γ4 DAN activation and sucrose also prefer the red-light-paired odour at testing compared with genetic and protocol controls (*n* = 14, 14, 12, 14, 14, 12). Different letters above bars in **b**, **c**, **d**, **f**, **g** indicate significantly different groups (*p* < 0.05; one-way ANOVA then Tukey’s HSD). **h**, Left: Protocol. Right: Coactivation of γ5n DANs with β′2&γ4 DANs during artificial training reduces CS+ approach performance irrespective of deprivation state (*n* = 12). Different letters above bars indicate significantly different genotypes (*p* < 0.05; two-way ANOVA then Tukey’s HSD; main effect of genotype: *F*(2,99) = 104.9, *p* < 0.0001). All data mean ± SEM; dots are individual data points that correspond to independent behavioural experiments. Exact statistical values and comparisons in Supplementary Information.

Another hallmark of unconstrained reward seeking is the concurrent neglect of physiological needs^50^. To test for need-indifferent reward seeking, we trained food-deprived *UAS-CsChr; 0273-GAL4* flies as before (Fig. 1c) by pairing a CS+ odour with nothing (mock training), optogenetic neuron activation, or dried sucrose. During testing, flies were given a choice for 120 s (which elicits similar performance as a testing period of 60 s; Extended Data Fig. 6a) between a T-maze arm with the CS− odour lined with dried sucrose and an arm with the CS+ odour lined with filter paper (Fig. 4a). Mock-trained flies showed preference for the sucrose-laden CS− tube, whereas sucrose-trained flies distributed evenly between the sucrose-laden CS− and sucrose-predicting CS+ tubes. By contrast, artificially trained food-deprived *UAS-CsChr; 0273-GAL4* flies exhibited a strong preference for the reward-predicting CS+ tube (Fig. 4a), despite food availability in the other T-maze arm. Thus, hungry flies appear to seek *0273*-neuron reward rather than feeding on sucrose.

Although some of the approximately 90 PAM DANs labelled by *R58E02-GAL4* are necessary for odour–sugar learning^15,22,23^, food-deprived *UAS-CsChr; R58E02-GAL4* flies and *UAS-CsChr/TH-GAL80; 0273-GAL4* flies artificially trained with red light both showed reduced preference for the reward-predicting CS+ over the sucrose-laden CS− compared with similarly trained *UAS-CsChr; 0273-GAL4* flies (Extended Data Fig. 6b). Thus, DANs targeted by *TH-GAL80* (and not labelled in sufficient number by *R58E02-GAL4*) appear to be required for *0273*-neuron-driven reward seeking to outcompete the availability of food.

We next tested whether *R48B04* and β′2&γ4 PAM DANs that produced persistent reward seeking despite punishment (Fig. 2d) also induced neglect of food. Flies expressing CsChr in *R48B04* DANs or β′2&γ4 DANs were trained by pairing CS+ odour with red light, then tested for choice between a paper-lined CS + T-maze arm and a sucrose-lined CS− arm. Both genotypes showed strong preference for the reward-predicting CS+ (Fig. 4b and Extended Data Fig. 6c). We verified that optogenetic training did not form a CS− odour-avoidance memory that might direct flies away from sucrose (Extended Data Fig. 6d) or potentiate learning with sugar (Extended Data Fig. 6e). *R48B04* and β′2&γ4 DAN-induced appetitive memories therefore also produce reward seeking at the expense of feeding.

We also tested whether prediction of β′2&γ4 DAN reward was preferred to prediction of sucrose. Flies trained with one odour paired with red light activation of *0273* neurons or β′2&γ4 DANs and another odour paired with sucrose preferred the previously red light-paired odour at testing, irrespective of the presentation sequence during training (Extended Data Fig. 6f,g). Thus, artificial β′2&γ4 DAN activation attaches greater expected reward value to an odour than that conferred by a natural reward such as sucrose.

Since persistent reward seeking despite shock could be partly attributed to decreased processing of aversion (Fig. 3d,e), we reasoned that artificial reward seeking despite available food might arise from reduced interest in sucrose, such as that originating from a concurrent satiety signal. We therefore tested whether β′2&γ4 DANs could directly or indirectly provide satiety-like ‘demotivational signals’ by CsChr-activating *0273*, *R48B04* (PAM-β′2, γ4 and γ5n), *R48B04-GAL4* and *R15A04-GAL80* (PAM-β′2&γ4) and *VT006202* (all PAM-γ5) neurons in naive food-deprived flies before testing their choice between a T-maze arm with blank paper and another containing paper with dried sucrose (Fig. 4c). In all genotypes that included expression in β′2&γ4 DANs (not those expressing in only γ5 DANs), prior CsChr-mediated neuronal activation decreased the number of flies accumulating in the sucrose arm compared with flies of the same genotype without optogenetic activation. These results are consistent with β′2&γ4 DANs (with or without concurrent γ5n DAN activity) conveying a teaching signal that motivates food-deprived flies to seek an odour predicting reward in addition to a satiety-like signal that devalues subsequent sucrose seeking.

Finally, we tested whether activation of γ5n DANs in parallel with the β′2&γ4 DAN teaching signal affected appetitive short-term memory (STM). Starved, dehydrated or satiated flies were trained by pairing odour with artificial activation of β′2, γ4 and γ5n DANs (*R48B04-GAL4*) or only β′2&γ4 DANs (*R15A04-GAL80; R48B04-GAL4*) (Extended Data Fig. 6h). β′2&γ4 DANs consistently reinforced robust state-independent appetitive STM, but coactivation of γ5n DANs with β′2&γ4 DANs decreased learned odour approach in every physiological state. Therefore, the full potential of β′2&γ4-mediated reward is restrained by concurrent activation of γ5n DANs. Since the activation of γ5 DANs in isolation does not reinforce aversive or appetitive learning (Extended Data Fig. 4a), we propose that γ5n DANs convey auxiliary teaching and satiety-like signals that modulate learned performance only in the presence of β′2 or γ4 DAN signals.

## Diverse and heterogeneous input

**Fig. 5:**
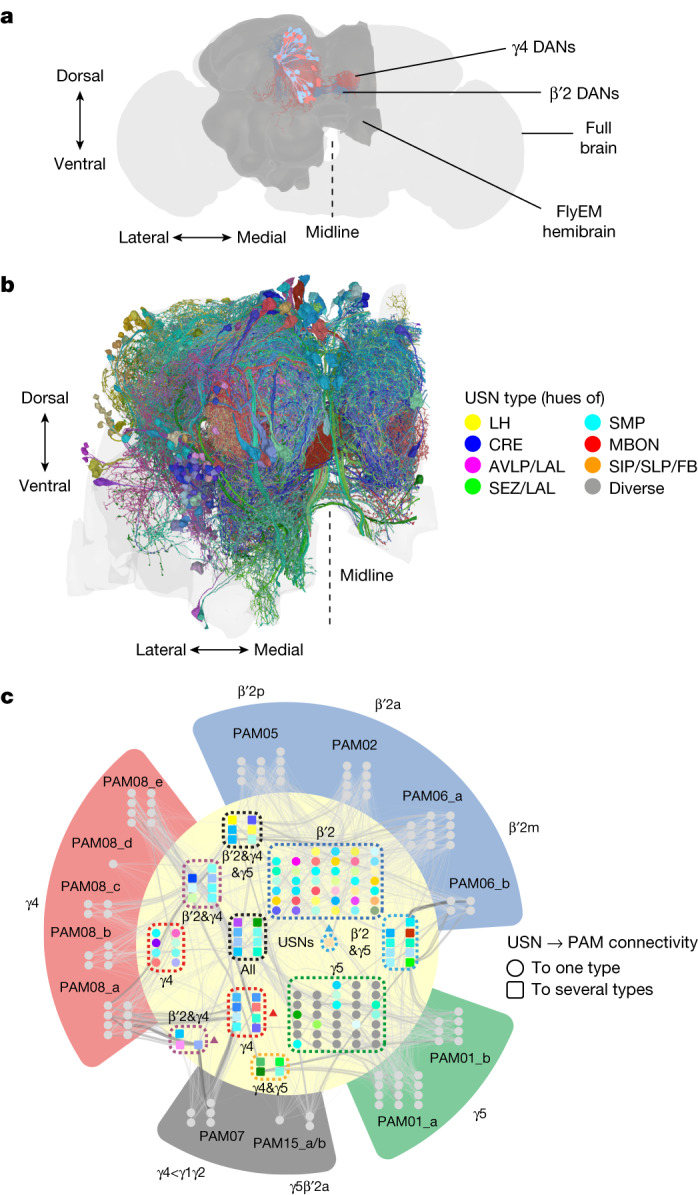
Reward DANs receive diverse and heterogeneous input. **a**, Volumetric reconstructions of β′2 DANs (PAM02, PAM05 and PAM06 (blue)) and γ4 DANs (PAM08 (red)) within the FlyEM hemibrain (dark grey) overlaid on a complete standard fly brain (light grey). **b**, Frontal view of volumetric reconstructions of the 402 USNs constituting the top 200 most strongly connected clusters to β′2 and γ4 DANs. USNs are rendered in hues of colours according to neurite location and are shown within the hemibrain neuropil (grey). Additional orientations and reconstructions of all 1,718 USNs connected to β′2 and γ4 DANs are presented in Extended Data Fig. 7 and Supplementary Video 1. **c**, Network diagram of USNs (inner cream circle) to β′2, γ4 and γ5 DANs (outer wedges) reveals a highly parallel input structure (thresholded at 0.4% of dendritic inputs for visibility). Individual DANs in outer wedges (grey circles) are grouped according to their compartments and types (different coloured wedges) and by subtype. USN clusters are grouped by connectivity pattern (dotted outlines) and connectivity to either one DAN type (circles) or multiple DAN types (squares) is denoted. Outlined USN clusters are labelled with corresponding DAN type targets; triangles mark USN groups that also innervate γ4<γ1γ2 DANs. USN node colours match those in **b**. Connector weight and transparency represents the percentage of dendritic input to these DANs provided by that USN (range 0.4% to 12.17%). See Extended Data Fig. 8 for a non-thresholded connectivity heat map and Supplementary Tables 1 and 2 and Methods for all connectivity information.

Each of the β′2, γ4 and γ5 DAN types contains multiple neurons^13^: β′2 (β′2a, 8 neurons; β′2m, 15 neurons; and β′2p, 10 neurons), γ4 (26 neurons; and γ4<γ1γ2, 5 neurons) and γ5 (19 neurons; and γ5β′2a, 3 neurons). Recent electron microscopy datasets of the *Drosophila* brain have revealed that smaller subsets within each DAN type are distinguishable by their unique synaptic input structures^13,21^. Specific groupings of individual DANs within the β′2, γ4 and γ5 types also receive common input from particular upstream neurons (USNs), including some implicated in representing the taste of sugar^13,21^. To understand how rewards might be represented by activation of *R48B04* DANs, we characterized all the USNs to β′2, γ4 and γ5 DANs using the complete connectome of the adult female fly hemibrain electron microscopy volume^51^ (Fig. 5a).

**Extended Data Fig. 7:**
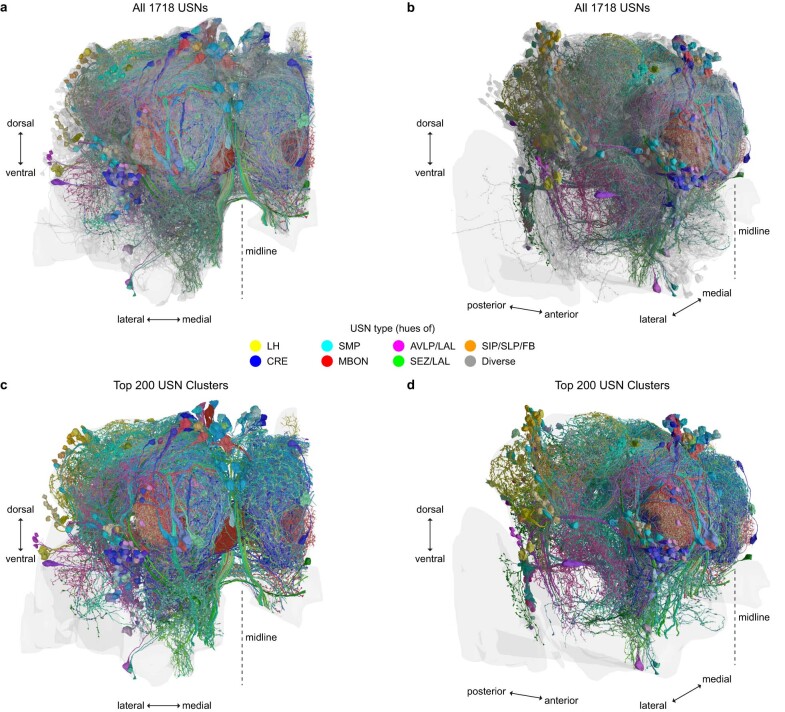
Inputs to β′2 and γ4 DANs represent a wide variety of information from across the brain. **a**, Frontal view of volumetric reconstructions of all 1718 upstream neurons (USNs) to β′2 and γ4 DANs from across the brain shown within the hemibrain neuropil (grey). The 402 USNs constituting the top 200 most strongly connected clusters to β′2 and γ4 DANs are rendered in hues of colours according to neurite location and all other USNs are grey. **b**, Latero-frontal view of the same 1718 USNs. **c**, Frontal view of the 402 USNs constituting the 200 most strongly connected clusters to β′2 and γ4 DANs (same as Fig. 5b; reproduced here for comparison). **d**, Latero-frontal view of the same 402 USNs. See Supplementary Video 1 for additional orientations and Supplementary Table 2 for all connectivity information.

We identified 1,996 USNs (excluding mushroom body Kenyon cells, PAM DANs, PPL DANs and other neurons;  Methods and Supplementary Table 1) providing dendritic input to β′2, γ4 or γ5 DANs (86 DANs in total), a 20-fold fan-in convergence of neuron number. In total, 1,718 of these USNs provided dendritic input to β′2 or γ4 DANs or both (Supplementary Table 2; visualized in Extended Data Fig. 7a,b and Supplementary Video 1). We next clustered the USNs into morphologically similar groups comprising 1 to 34 neurons and visualized the 200 clusters that were most strongly connected to β′2 or γ4 DANs or both (402 neurons visualized in Fig. 5b, Extended Data Fig. 7c,d and Supplementary Video 1). These USN clusters emanate from multiple brain regions.

**Extended Data Fig. 8:**
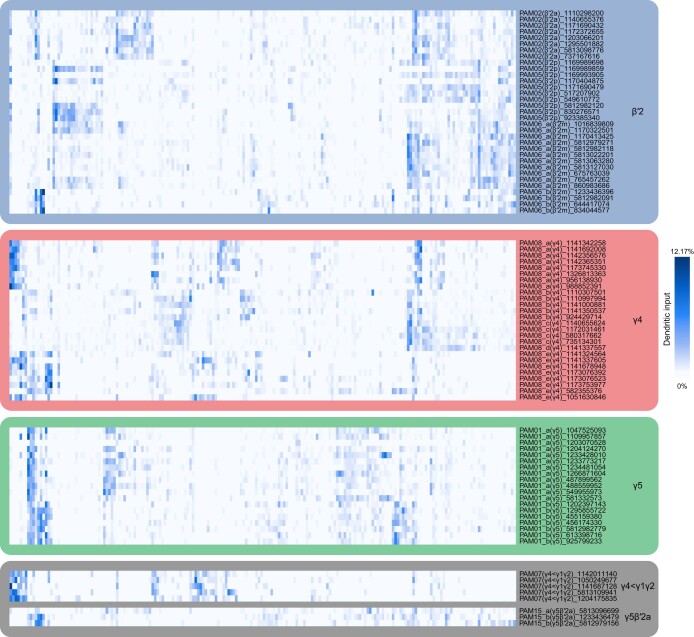
Heatmap of inputs to β′2, γ4, and γ5 DANs. Connectivity heatmap of the 200 strongest upstream neuron (USN) clusters (comprising 450 USNs) to β′2 (PAM02, PAM05, PAM06) DANs, γ4 (PAM08) DANs, γ5 (PAM01) DANs, γ4<γ1γ2 (PAM07) DANs, and γ5β′2a (PAM15) DANs in the right hemisphere of the FlyEM hemibrain. The USN clusters group together and reveal an elaborate parallel structure through their connectivity to single or multiple DAN subtypes. Values represent the percentage dendritic input from individual USN clusters (columns) to individual DANs (rows). All connectivity information is available in Supplementary Table 1.

We separately analysed connectivity patterns of the top 200 input clusters to β′2, γ4 or γ5 DANs (450 neurons in total) (Fig. 5c and Extended Data Fig. 8; see Methods for details of thresholds applied). Connectivity to DANs is highly parallel, with many DAN types and even subtypes receiving input from particular USN clusters. For example, 40 USN clusters connected to β′2 DANs but not γ4 and γ5 DANs, 8 clusters connected to γ4 DANs, and 33 clusters connected to γ5 DANs (Fig. 5c). Twenty-four USN clusters provided shared monosynaptic input to both β′2 and γ4 DANs, of which 14 also connected to γ5 DANs (Fig. 5c). It is noteworthy that only two of these 24 shared input clusters to β′2 and γ4 DANs are suboesophageal zone (SEZ) output neurons, which convey gustatory sensory information from the SEZ^21^. In addition, we found the octopaminergic neuron VPM4, which suppresses persistent odour-tracking behaviour^52^ and promotes sugar feeding^53^ to be connected to γ4 and γ4<γ1γ2 DANs.

**Extended Data Fig. 9:**
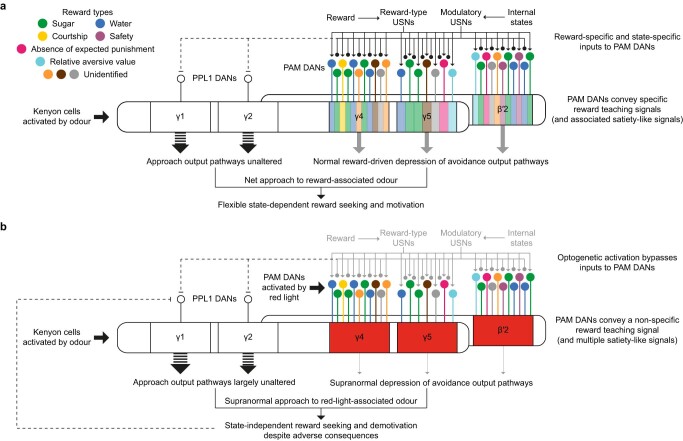
Artificial activation of β′2, γ4, and γ5 DANs simultaneously conveys multiple reward types and satiety-like signals. **a**, In healthy flies, β′2, γ4, and γ5 PAM DANs are activated by specific reward-type upstream neurons (USNs, with excitatory arrow inputs) and modulated by modulatory USNs (with modulatory circle inputs). Some modulatory USNs may also inhibit PPL1 DANs that convey aversive punishment to the γ1 and γ2 compartments^29,30^. The PAM DANs are coloured according to the rewards they may represent according to prior studies^21,23–28,45^ in addition to rewards that have not yet been identified. When a healthy fly encounters a reward in a presence of an odour, the reward activates only specific reward-type USNs, whose activation of PAM DANs is concurrent with modulatory input from USNs conveying information about the corresponding physiologically relevant internal state. The PAM DANs therefore convey specific reward teaching signals (and associated satiety-like signals) in a state-appropriate manner to mushroom body Kenyon cells that are coincidentally activated by the odour, leading to normal reward-driven depression of avoidance output pathways. Since approach pathways from the mushroom body are unaffected by reward signalling, flies subsequently demonstrate a net approach to the reward-associated odour, enabling flexible state-dependent reward seeking and motivation. **b**, In flies whose β′2, γ4, and γ5 PAM DANs express CsChr and are activated directly by red light, information about specific reward types and physiologically relevant internal states is disregarded. The PAM DANs convey a non-specific reward teaching signal consisting of the combined value of multiple rewards (and multiple satiety-like signals) to Kenyon cells, leading to supranormal depression of avoidance output pathways. Flies subsequently demonstrate supranormal approach to the red-light-associated odour, resulting in state-independent reward seeking and demotivation to other natural rewards. PPL1 DANs that convey aversive punishment to the γ1 compartment also consequently undergo indirect inhibition, which manifests when flies display punishment-resistant reward seeking.

Since several nutrition-related^23,24,39^ and nutrition-independent types of reward^21,25–28,45^ have been determined to involve unique subsets or combinations of β′2, γ4 and γ5 DANs, it is conceivable that a variety of other unknown rewards will also be conveyed to these DANs through their elaborate highly parallel USN input structure (Fig. 5c). We therefore propose that artificial activation of *R48B04* or only β′2 and γ4 DANs simultaneously conveys the value of multiple types of reward (Extended Data Fig. 9).

## Motivational control of DAN responses

The formation and expression of memories reinforced by sugar and water are dependent on the relevant states of hunger and thirst^24,29,30^, demonstrating a tight link to physiological needs. Different β′2 and γ4 DANs have been implicated in state-dependent reinforcement of sugar^23^ or water memory^24^, in controlling food or water seeking^24,31^, and in state-relevant expression of food-seeking and water-seeking memories^30^. Inducing unconstrained seeking of reward with these neurons thus seems likely to involve bypassing this complex level of motivational control (Extended Data Fig. 9).

**Fig. 6:**
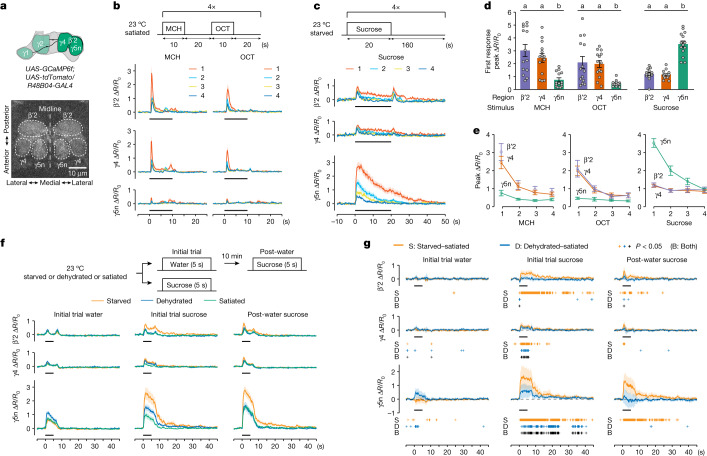
Physiological state-dependent control of DAN responses. **a**, Schematic (top) and two-photon imaging of regions of interest (ROI) (bottom) for DANs co-expressing GCaMP6f (displayed) and tdTomato. **b**, Top, odour presentation protocol. Bottom, calcium responses in β′2 (top), γ4 (middle) and γ5n DANs (bottom) to each MCH (left) and OCT (right) presentation. Black bars throughout indicate stimulus application. **c**, Top, sucrose presentation protocol. Bottom, DAN responses to each sucrose presentation. **d**, Peak heights of first DAN responses to MCH, OCT or sucrose. β′2 and γ4 DANs exhibit larger odour responses whereas γ5n DANs have larger sucrose responses. The break in the *x* axis demarcates separate experiments. Different letters above bars indicate significantly different regions (*P* < 0.05; two-way repeated measures ANOVA for each odour or one-way repeated measures ANOVA for sucrose then Tukey’s HSD). **e**, Only γ5n DAN peak responses diminish with repeated sucrose presentations. Data in **d**,**e** are mean ± s.e.m.; dots are individual data points that correspond to individual flies. **f**, Top, feeding protocol. Bottom, responses of β′2 (top), γ4 (middle) and γ5n DANs (bottom) to initial-trial water (left), initial-trial sucrose (middle) or post-water sucrose (right) in starved, dehydrated or satiated flies. **g**, Mean difference curves for responses in starved or dehydrated flies versus satiated flies. Crosses indicate significantly different recording frames (*P* < 0.05; two-sided unpaired *t*-test, not corrected for multiple comparisons). S, starved − satiated; D, dehydrated − satiated; B, common to S and D. Response curves show mean ± s.e.m. (**b**,**c**,**f**) or mean difference ± 95% confidence interval (**g**) for the normalized ratio of GCaMP6f to tdTomato signal (Δ*R/R*_0_); presentation numbers or physiological states are denoted by curve colour. *n* = 14 flies (**b**–**e**) and *n* = 24 flies (**f**,**g**). Exact statistical values and comparisons are presented in Supplementary Information.

To directly test whether *R48B04* DANs are sensitive to motivational state, we used two-photon in vivo calcium imaging to characterize physiological responses of β′2, γ4 and γ5n DANs when flies were exposed to the odours used in conditioning or fed sucrose. Head-tethered *R48B04-GAL4* flies expressing GCaMP6f were given repeated presentations of odour or sucrose. Co-expression of the red fluorescent protein tdTomato provided a reference for sample movement. Activity in β′2, γ4 and γ5n DAN presynaptic arbours was recorded simultaneously in the same imaging plane and signals were anatomically demarcated for independent analyses^22,24^ (Fig. 6a), enabling comparisons both within and between individual flies. Satiated flies were presented with 4-methylcyclohexanol (MCH) then 3-octanol (OCT) alternately 4 times for 10 s each with an inter-odour interval of 20 s (Fig. 6b). Alternatively, starved flies were presented with a droplet of 1 M sucrose 4 times for 20 s each with an inter-feeding interval of 160 s (Fig. 6c).

Each odour or sucrose presentation evoked a substantial increase from baseline in all DAN classes (Fig. 6b–e). However, β′2 and γ4 DANs responded most strongly to odours, whereas γ5n DANs responded most strongly to sucrose (Fig. 6b–e). β′2 and γ4 DANs exhibited noticeable off-responses to sucrose presentation (Fig. 6c), consistent with providing a stimulus-locked teaching signal. By contrast, γ5 DAN sucrose responses lasted beyond the sucrose presentation and exhibited progressively decreasing peak responses on repeated exposures (Fig. 6c,e). Differential responses to odours and sucrose thus support our behavioural findings that β′2&γ4 DANs and γ5n DANs convey different signals during appetitive olfactory conditioning.

Previous imaging studies have reported that DANs innervating the β′2, γ4 or γ5 compartments respond to consumption of water in dehydrated flies^24^ and of sucrose in starved flies^15,22,42,45^. However, although an aqueous solution provides control over the onset and offset of feeding a tethered fly, the water solvent and sucrose solute are both likely to contribute to ‘sugar’ *R48B04* DAN responses. We therefore designed experiments to differentiate between water-specific and sucrose-specific DAN responses in different physiological states.

We recorded GCaMP6f fluorescence in *R48B04-GAL4* flies that were starved, dehydrated or provided with ad libitum access to food and water (that is, satiated) (Fig. 6f). Flies from each group were given either water or 1 M sucrose for 5 s (termed ‘initial-trial water’ or ‘initial-trial sucrose’ respectively). Ten minutes later, flies in the initial-trial water group were given a 5 s presentation of 1 M sucrose (termed ‘post-water sucrose’). Comparing initial-trial sucrose and post-water sucrose responses controls for the presence of water and reveals state-dependent response components specific to water and sucrose (Fig. 6f).

Conversely, identifying similarities across all feeds and all physiological states reveals response components that are common to feeding and are likely to be state-independent. Water and sucrose solution each evoked an increased calcium response relative to baseline for each *R48B04* DAN subtype (β′2, γ4 and γ5n) in all three physiological states (Fig. 6f). These signals may therefore represent the general salience of feeding or feeding-related motor signals^45^.

**Extended Data Fig. 10:**
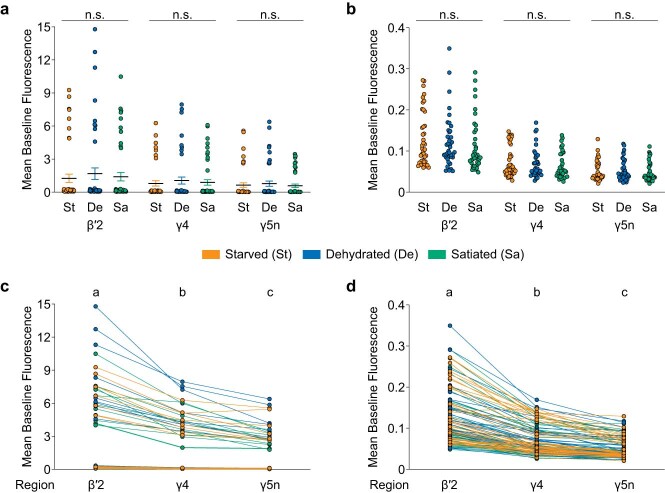
Manipulations of physiological state do not affect the baseline calcium signals of *R48B04* DANs. **a**, The mean baseline fluorescence (for the 60 s before stimulus presentation relative to the mean of a non-implicated region) of the initial-trial water and initial-trial sucrose samples in Fig. 6f for β′2, γ4, and γ5n DANs (*n* = 48 flies per state). All data mean ± SEM. **b**, Distribution of samples with mean baseline fluorescence below 0.4 (81.25% of all samples) for clarity. **c**, Each region has a different mean baseline fluorescence irrespective of physiological state (*n* = 144 flies in total from three states). Each data point from each region is connected to two data points corresponding to the other two regions of the same fly. **d**, Comparison across regions for 81.25% of samples with mean baseline fluorescence below 0.4 for clarity. Dots are individual data points that correspond to individual flies. All data points and connecting lines are coloured according to the physiological state of each fly (Starved: St, orange; Dehydrated: De, blue; or Satiated: Sa, green). No differences in mean baseline fluorescence are observed across physiological states in **a**, **b** (two-way ANOVA; main effect of state *F*(2,141) = 0.2188; *p* = 0.8038; n.s. = not significant). Different letters above groups in **c**, **d** indicate significantly different regions (*p* < 0.05; two-way repeated measures ANOVA then Tukey’s HSD; main effect of region *F*(2,282) = 29.23; *p* < 0.0001). Exact statistical values and comparisons in Supplementary Information.

To determine deprivation-state-specific water and sucrose responses, we calculated mean difference curves between responses in each deprivation state and those in the satiated state (Fig. 6g). We found that starvation (Fig. 6g, orange) elevated responses for all three *R48B04* DAN subtypes to initial-trial sucrose and post-water sucrose but not to initial-trial water. Thus starvation specifically increases *R48B04* DAN responses to physiologically relevant and satiating sucrose. In comparison, dehydration (Fig. 6g, blue) elevated responses to initial-trial water for γ5n DANs only to initial-trial sucrose for γ4 and γ5n DANs, but not to post-water sucrose in any DAN subtype. Dehydration thus specifically increases γ4 and γ5n DAN responses to the consumption of physiologically relevant and satiating water. Finally, we verified that manipulations of physiological state did not affect the baseline calcium signals of *R48B04* DANs (Extended Data Fig. 10).

Together, our results suggest that β′2 DAN responses are modulated specifically by starvation, γ4 DAN responses are modulated by dehydration, and γ5n DAN responses are modulated by both starvation and dehydration. We therefore propose that thirst and hunger states constrain the activation of specific subsets of *R48B04* DANs to convey coordinated and appropriate reward teaching and satiety-like signals when a fly ingests state-relevant water or sucrose. State-dependent gating in healthy flies ensures that the relevant DAN signals motivate appropriate need-directed seeking rather than punishment-resistant and need-indifferent reward seeking (Extended Data Fig. 9).

## Discussion

Here we have demonstrated dopaminergic mechanisms that generate cued reward seeking despite adverse consequences in *Drosophila*. Using *Drosophila* enabled identification of specific DAN populations whose synthetic activation during a single training session recapitulates (albeit on a timescale of minutes and without many repetitions) some of the phenotypes resembling ‘compulsive-like’ behaviour^3,54^ seen in mice trained over days with many more experiences of synthetic reward^7^. The relevant fly DAN populations have a highly heterogeneous input structure^13,21^ consistent with differential representations of various types of reward^15,16,21,24–28^. We note that ethanol—a substance that reinforces learning that produces shock-resistant reward seeking in flies^33^—activates a broad population of PAM DANs^40^. It will be interesting to investigate how DAN dysfunction could lead to unconstrained seeking of specific rewards such as alcohol^33^ or sugar^55^ over extended periods of time, and especially whether there might be individual differences in susceptibility^56^ in flies.

We show that cardinal features of reward seeking despite adverse consequences can arise from mechanisms besides the high incentive value of the expected—and perhaps multimodal—reward. Owing to opposing network connectivity within the DAN system, reward DAN activation indirectly impairs the function of aversive DANs, which manifests as ‘risk-taking’ of enduring shock while seeking reward. In addition, simultaneously engaging the heterogeneous reward DANs overwhelms and bypasses their normally precise state-specific and reward-specific gating (Extended Data Fig. 9). As a result, subsequent valuation of other resources is diminished, and starved flies forego food when cued to seek reward. Moreover, activation of mouse ventral tegmental area DANs can drive compulsive-like behaviour^7^, whereas their inhibition generates aversion^46,47^. The dopaminergic mechanisms described here that give rise to unconstrained seeking of reward in the fly may therefore be generally informative for understanding similar behavioural dysfunction in mammals.

## Methods

### Fly strains

Canton-Special flies^57^ were used as wild-type. Transgenes were expressed with GAL4 lines from the InSITE collection^58^, the Janelia FlyLight collections^44,59^ or the Vienna Tile collection^60^: *0273-GAL4*^16,58^, *R48B04-GAL4*^23,24^, *R58E02-GAL4*^15^, *R29C06-GAL4*^59^, *MB042B-GAL4*, *MB056B-GAL4*, *MB109B-GAL4*, *MB312C-GAL4*, *MB316B-GAL4* and *MB504B-GAL4*^44^, *VT006202-GAL4*^21,61^. *TH-GAL4* is from ref. ^62^. *GAL80* transgenes co-expressed with *0273-GAL4* are as follows: *Cha-GAL80*^63^, *GAD1-GAL80*^64^, *Vglut-GAL80*^65^ and *TH-GAL80*^66^. The optogenetic effectors *UAS-CsChrimson::mVenus* (*UAS-CsChr*)^67^ and *UAS-GtACR1*^68^ were expressed under the control of specific GAL4 drivers. The reporter *UAS-mCD8::GFP*^69^ was expressed with *0273-GAL4* together with various *GAL80* transgenes: *R15A04-GAL80*^24^, *R48B04-GAL80*^23^ and *R58E02-GAL80*^15^. *LexAop-rCD2::mRFP*^70^ was expressed with *247-LexA::VP16*^71^. The *R15A04-GAL80; R48B04-GAL4* combination has been described^24^. *tsh-GAL80* is from ref. ^43^. For two-photon imaging experiments *20XUAS-IVS::GCaMP6f*^72^ and *UAS-myr::tdTomato*^73^ were expressed under the control of *R48B04-GAL4*.

### Fly husbandry

All *D. melanogaster* strains were maintained at 25 °C and 60% humidity in a 12:12 h light:dark cycle with light provided between 8 AM and 8 PM. For all behavioural experiments, flies were reared on yellow cornmeal agar food containing deionized water, 7.2 g l^−1^ agar (Fisher Scientific), 25 g l^−1^ autolysed yeast extract (Brian Drewitt), 47.3 g l^−1^ cornmeal (Brian Drewitt), 100 g l^−1^ dextrose (d-glucose anhydrous, Fisher Scientific), 2.2 g l^−1^ tegosept (methyl 4-hydroxybenzoate) (Sigma-Aldrich), and 8.4 ml l^−1^ ethanol (Sigma-Aldrich). All cornmeal agar food was prepared by boiling, not autoclaving. Before deprivation for all optogenetic experiments, adult flies were reared in darkness for 3 days on yellow cornmeal agar food containing 1 mM all-*trans*-retinal (Sigma-Aldrich). For all physiological experiments, flies eclosed on brown cornmeal agar food containing deionized water, 6.75 g l^−1^ agar, 25 g l^−1^ yeast, 62.5 g l^−1^ cornmeal, 37.5 ml l^−1^ molasses (Brian Drewitt), 4.2 ml l^−1^ propionic acid (Fisher Scientific), 1.4 g l^−1^ tegosept and 7 ml l^−1^ ethanol; 0-to-2-day-old adult flies were then transferred to yellow cornmeal agar food.

### Food and water deprivation

Flies were aliquoted into groups of ~100 before behavioural experiments or ~10 before physiological experiments. For starvation, flies were food-deprived for 20 to 26 h in a 25 ml vial containing a 2 cm × 3 cm piece of filter paper with 1% agar at the base. Vials were stored at 22 °C throughout the starvation period. For dehydration, flies were kept in a 25 ml vial without water for 4 h to 6 h. Throughout the dehydration period, flies had access to a sheet of dry sucrose-coated filter paper (2 cm × 3 cm) resting on top of a layer of cotton wool, which separated the flies from a thick layer of the desiccant Drierite (calcium sulfate, Sigma-Aldrich) at the base. Vials were stored at 22 °C throughout the dehydration period in a sealed polystyrene box containing a similar arrangement of Drierite and cotton wool. Satiated flies—that is, flies provided with ad libitum access to food and water—were transferred into a 25 ml vial containing a 2 cm × 3 cm piece of filter paper with 1% yellow cornmeal agar food at the base and then stored at 22 °C for 20–26 h before experiments.

### T-maze olfactory behavioural experiments

Male flies from GAL4 lines were crossed to female flies from effector lines and their mixed-sex 4-to-12-day-old offspring were tested in groups of ~100 flies each for all T-maze behavioural experiments. The two odours used for testing in all olfactory experiments were MCH and OCT^57^ (Sigma-Aldrich). For Fig. 3c, the initial two odours used for optogenetic training were isoamyl acetate (IAA) and ethyl butyrate. Each odour was diluted ~1:10^3^, specifically, 10 µl MCH or 7 µl OCT or 10 µl IAA or 7.5 µl ethyl butyrate in 8 ml mineral oil (Sigma-Aldrich). All experiments were performed at 23 °C and 55% to 65% relative humidity.

### Olfactory conditioning with sucrose

For Figs. 1a,c and 4a, sucrose was prepared as a saturated solution (~400 g l^−1^), of which 3 ml was pipetted onto a 6 cm × 8 cm piece of filter paper. Excess solution was drained from the paper, which was then rolled into a tube and allowed to dry overnight. Appetitive training with sucrose was conducted as previously described^74^. Groups of flies were first transferred to a training tube of a T-maze lined with filter paper and then exposed to an odour (the CS−) for 2 min. Following 30 s of clean air in the same tube, flies were transferred to another tube lined with dried sucrose then immediately exposed to another odour (the CS+) for 2 min. Mock-trained flies in Figs. 1c and  4a experienced the same protocol as sucrose-trained flies, except that the second odour tube was also lined with filter paper instead of dried sucrose. Both mock-trained and sucrose-trained flies were transferred to the T-maze elevator after CS+ exposure and immediately given a choice between the CS− and CS+ odours.

### Olfactory conditioning with optogenetic activation

For Figs. 1b,c, 2c,d and 4a,b, olfactory conditioning with optogenetic neural activation was conducted as previously described^21^. Groups of flies were transferred into a tube on which three red LEDs (700 mA, centred at 630 nm, 3 W maximum power; Multicomp) were mounted. Flies were exposed to the CS− odour for 2 min, followed by 30 s of clean air, then 2 min of exposure to the CS+ odour paired with 500 Hz red light (1 ms pulses driven at 1.25 V with 0.1 ms delay). Red light was pulsed under the control of a DG2A Train/Delay Generator (Digitimer) coupled with a DS2A Isolated Constant Voltage Stimulator (Digitimer). For the experiments in Figs. 1c and  4a, flies were transferred after the clean air to another tube (in which they were exposed to the CS+); whereas for Figs. 1b,  2c–e and  4b, flies were exposed to each odour in the same tube without any transfers. Red-light-trained flies were transferred to the T-maze elevator after CS+ exposure and immediately given a choice between the CS− and CS+ odours.

For the experiments in Extended Data Fig. 1c,e, flies were exposed to the CS+ odour simultaneously paired with 90 or 120 V pulsed electric shocks (of 1.5 s duration each at 0.2 Hz) and red light, whereas for the experiment in Extended Data Fig. 6e, flies were exposed to the CS+ odour simultaneously paired with both sucrose and red light. For the experiments in Extended Data Fig. 6f,g, flies were exposed to one odour paired with sucrose and another odour paired with red light.

### Olfactory conditioning with optogenetic inhibition

For Fig. 3a,b, olfactory conditioning with optogenetic inhibition was conducted as for optogenetic activation described above, with the following differences. Groups of flies were initially transferred to a tube on which three green LEDs (700 mA, centred at 530 nm, Multicomp) were mounted. Flies were exposed to the CS− odour for 2 min, followed by 30 s of clean air, then transferred to another tube in which they were exposed for 2 min to the CS+ odour paired with continuous green light. Green-light-trained flies were transferred to the T-maze elevator after CS+ exposure and immediately given a choice between the CS− and CS+ odours.

### Sequential optogenetic then aversive olfactory training

For Fig. 3c, flies were first trained with optogenetic activation (without transfers between tubes) and IAA and ethyl butyrate as described above. After being exposed to fresh air for another 30 s following the CS+ exposure, they were transferred to another tube for aversive olfactory training with electric shock and two different odours (MCH and OCT). Aversive olfactory training was performed as previously described^75,76^. Groups of flies were transferred to a tube lined with a conductive copper coil. Electric shocks were delivered under the control of an S48 square pulse stimulator (Grass Technologies). Flies were exposed for 1 min to the CS+ odour paired with 12 shocks (90 V pulses of 1.5 s duration at 0.2 Hz), then 45 s of fresh air, followed by 1 min exposure to the CS− odour without shock, all in the same tube. Flies were transferred to the T-maze elevator after CS− exposure then immediately given a choice between the CS− and CS+ odours used during the aversive training (not the odours used during the first round of red-light training). For the experiment in Extended Data Fig. 4e, flies were trained with only the odours MCH and OCT.

### T-maze olfactory testing with simultaneous shock

For Figs. 1a–c and 2c–e, after appetitive olfactory training, flies were permitted to choose in darkness between two tubes (both lined with conductive copper coils) that contained either the CS+ odour electrified with 90 V shocks (of 1.5 s duration each at 0.2 Hz) or the non-electrified CS− odour. Testing duration for Figs. 1a,b and  2d, varied at increasing intervals (15 s, 30 s or 60 s), whereas for Figs. 1c and  2c,e, testing duration was fixed at 60 s. For the variable-test-duration experiments (Figs. 1a,b and 2d), the leftmost treatment group (per genotype) is a positive control in which flies experienced appetitive training but neither tube was electrified during testing, whereas the rightmost treatment group (per genotype) is a negative control in which flies experienced mock training and the CS+ odour was electrified during testing. For the experiment in Extended Data Fig. 4d, flies were separated after testing depending on their choice of entering either the electrified CS+ or non-electrified CS − T-maze arm during the first test and then retested 30 s later with the same choice.

Performance index (PI) was calculated as the number of flies in the CS+ tube minus those in the CS− tube, divided by the total number of flies. Flies that entered each tube were transferred into separate vials and immobilized by freezing to permit counting. To account for any odour bias, a single PI score was calculated from the mean scores of two independent experiments in which separate groups of flies of the same genotypes were trained with reciprocal combinations of MCH and OCT as the CS+ and CS− odours.

### T-maze olfactory testing without simultaneous stimuli

For Fig. 3a–c, after olfactory training, flies were allowed to choose in darkness for 2 min (1 min for Fig. 3b) between two tubes (not lined with filter paper) that contained either the CS− or CS+ odours. Testing PI was calculated as described above.

### T-maze olfactory testing with simultaneous sucrose

For Fig. 4a,b, after appetitive olfactory training, flies were allowed to choose in white light for 2 min between two tubes that contained either the CS− odour presented with a dried sucrose paper or the CS+ odour presented with a blank filter paper. Testing PI was calculated as described above. For the experiment described in Fig. 4b, the leftmost treatment group is a positive control in which flies experienced optogenetic training but both tubes were lined with blank filter paper during testing, whereas the rightmost treatment group is a negative control in which flies experienced mock training and the CS− odour was presented with dried sucrose paper during testing.

### Shock avoidance experiments

For Fig. 3d, flies were transferred to an odourless tube (not lined with filter paper) on which three red LEDs were mounted. Flies were exposed to 500 Hz red light for 2 min, then transferred to the T-maze elevator and immediately allowed to choose in darkness for 1 min between two copper-lined tubes, one of which delivered 90 V electric shocks of 1.5 s duration at 0.2 Hz. Control groups were not exposed to red light before testing. For the naive shock avoidance experiment in Fig. 3e, flies were directly transferred to the T-maze elevator without training and immediately allowed to choose in darkness between two copper-lined tubes (each mounted with three green LEDs), one of which was coupled with 90 V shocks. Both tubes were illuminated with continuous green light throughout testing. For both experiments, the preference index (PI) was calculated as the number of flies in the shock tube minus those in the control tube, divided by the total number of flies. Each experiment contributed a single PI value (rather than the mean scores of two experiments), but the tube conducting electric shocks alternated between experiments.

### Sucrose approach experiments

For Fig. 4d, flies were transferred to an odourless tube (not lined with filter paper) on which three red LEDs were mounted. Flies were exposed to 500 Hz red light for 2 min, then transferred to the T-maze elevator and immediately allowed to choose for 2 min in white light between two tubes, one laden with dried sucrose paper and the other with control filter paper. Control groups were not exposed to red light before testing. The preference index (PI) was calculated as the number of flies in the sucrose tube minus those in the control tube, divided by the total number of flies. Each experiment contributed a single PI value (rather than the mean scores of two experiments), but the tube containing sucrose changed sides between experiments.

### T-maze olfactory testing for individual CS+ and CS− memories

For Extended Data Fig. 6d, to isolate the individual CS+ and CS− memories, a novel odour (16 µl IAA in 8 ml mineral oil) was used to replace either the CS+ or CS− odour during training. When testing for CS+ odour memory, MCH or OCT were used as the CS+ odour for half of the reciprocal training experiments each, whereas IAA was always used as the CS− odour. When testing for CS− odour memory, MCH or OCT were used as the CS− odour for half of the reciprocal training experiments each, whereas IAA was always used as the CS+ odour. The testing odours were always MCH and OCT for all treatment groups.

### Single-cell RNA sequencing

Central brains were dissected from 32 *UAS-CsChr; 0273-GAL4* flies and a cell suspension was generated as described^77^. Single cells were then encapsulated with oligonucleotide barcoded gel beads in nanolitre volumes using the Chromium Single Cell 3′ Reagent Kit v3 (10x Genomics)^78^, following the manufacturer’s instructions. Following in-droplet reverse transcription, droplets were lysed and cDNA libraries were amplified and sequenced on an Illumina HiSeq4000 platform. After filtering out low-quality barcodes and putative cell doublets, we retrieved a total of 11,502 cells with an average of 5,673 unique molecular identifiers (UMIs) detected per cell. Barcoded sequencing reads were aligned to the *D. melanogaster* reference genome (release 6.25 from https://flybase.org/) and to the *UAS-CsChr* transgene (GenBank KJ995863.2)^67^. We considered only genes that were present in at least 10 cells, resulting in 9,935 genes (including the *UAS-CsChr* transgene) being detected. UMAP reduction^79^ of the data and clustering was performed using the Seurat v3 R package^80^. *CsChr*-expressing cells were classified into neurotransmitter types based on their relative expression of *Vmat* and *DAT* for dopamine, *vesicular acetylcholine transporter* (*VAChT*) for acetylcholine, *glutamic acid decarboxylase 1* (*Gad1*) for GABA, and *vesicular glutamate transporter* (*VGlut*) for glutamate.

### Confocal imaging

*0273*-*GAL4* expression (with or without *GAL80* co-expression) was visualized using *UAS-mCD8::GFP*. For Fig. 2a, the mushroom body was co-labelled with *247-LexA::VP16*-driven expression of *LexAop-rCD2::mRFP*. Brains from 2-to-4-day-old flies were dissected in phosphate buffered saline (PBS: 1.86 mM NaH_2_PO_4_, 8.41 mM Na_2_HPO_4_, and 175 mM NaCl) and fixed in 4% v/v paraformaldehyde (PFA) for 20 min at room temperature. Fixed brains were washed in PBS and mounted onto a glass slide in Vectashield Antifade mounting medium (Vector Labs), covered with a glass coverslip, and sealed with nail varnish.

Native GFP and mRFP fluorescence was imaged using a Leica TCS SP5 X confocal microscope with an HCX PL APO 40× 1.3 N.A. oil-immersion objective (Leica). GFP was imaged with 20% laser power, whereas mRFP (when required) was imaged with 7% laser power. Laser power and acquisition settings remained constant for the imaging of all samples. The resolution of each image stack acquired was ~2 pixels per µm and the voxel size was ~0.45 µm × ~0.45 µm × 0.1678 µm. Acquired images were processed using Fiji^81^. Maximum intensity projections of *0273*-labelled neurons and mushroom bodies (when applicable) were created in Fiji for visualization. The number of *0273*-labelled PAM neuron cell bodies per hemisphere was counted manually in Fiji; each hemisphere was counted as a separate sample.

### Clustering of USNs by morphology

Synaptic connectivity data was obtained from the hemibrain nanoscale connectome electron microscopy dataset (v1.2.1, neuprint.janelia.org)^51^ via Python-based NAVis functionalities (v1.3.0)^82^. USNs with dendritic connectivity to β′2 (PAM02, PAM05 and PAM06), γ4 (PAM08), γ4<γ1γ2 (PAM07), γ5 (PAM01) and γ5β′2a (PAM15) DANs were defined as all input neurons. Kenyon cells, other PAM DANs, PPL DANs, anterior paired lateral neurons, dorsal paired medial neurons, and three mushroom body output neurons (MBONs) (MBON05 (γ4>γ1γ2), MBON11 (γ1pedc>α/β) and MBON09 (γ3β′1)) that provide axo-axonal input within the mushroom body neuropil were excluded. Generally, clustering of USNs was performed according to Neuprint type^51^. SEZ-associated neurons were clustered separately, as described^13,21^. In brief, MBDL1 and MBDL2 axon neurons of the left hemisphere were mirrored, and then clustered with right-hemisphere neurons by morphology (using Manhattan distance metrics and the average linkage criterion) to yield a fine cluster granularity using R-based natverse (https://natverse.org) functionalities^83^.

### Calculation and ranking of percentage dendritic input

The percentage dendritic input from each USN to each individual β′2 (PAM02, PAM05 and PAM06), γ4 (PAM08), γ4<γ1γ2 (PAM07), γ5 (PAM01) and γ5β′2a (PAM15) DANs was calculated as the fraction of total dendritic input (not including the neurons previously excluded) provided to the DAN by that USN. The sum of the percentage dendritic inputs of all USNs in each USN cluster (comprising 1 to 34 USNs) was then calculated to determine the strength of each cluster’s combined percentage dendritic input to the DANs. USN clusters were then ranked by the strength of their percentage dendritic input to either all seven β′2, γ4, and γ5 DAN types (for the connectivity map and heat map) or the four β′2 and γ4 DAN types (for the volumetric reconstructions).

The top 200 clusters providing the strongest dendritic input were then selected for further analysis. For dendritic input to all seven β′2, γ4, and γ5 DAN types, the top 200 clusters comprised 450 neurons, whereas to only the four β′2 and γ4 DAN types, the top 200 clusters comprised 402 neurons. 163 clusters (and 339 USNs) are shared between the two sets of top 200 clusters. 37 clusters (111 USNs) are unique to the top 200 strongest inputs to all seven β′2, γ4, and γ5 DAN types, whereas another 37 clusters (63 USNs) are unique to the top 200 strongest inputs to only the four β′2 and γ4 DAN types. All connectivity data are available in Supplementary Tables 1 and 2.

### Volumetric reconstructions

For Fig. 5b, 3D meshes of neuronal volumetric reconstructions were obtained from the hemibrain electron microscopy dataset v1.2.1 via Python-based NAVis v.1.3.0 functionalities^82^. Composition and rendering of three-dimensional projections and Supplementary Video 1 were created using Blender v.3.2.2 (Blender Foundation), into which data were imported via NAVis-Blender interface functionalities. Brain meshes were obtained from the R-based natverse package nat.flybrains v.1.7.4^83^. The top 200 USN clusters most strongly connected to β′2 and γ4 DANs were coloured manually in hues of colours according to neurite location.

### Input network connectivity map

For Fig. 5c, to display the input network map, a threshold of 0.4% dendritic input was applied for USN-cluster-to-DAN connections. Nodes representing DANs were grouped by Neuprint DAN subtype and nodes representing USN clusters were grouped by DAN connectivity (see below). The 0.4% threshold was determined by iterative testing of thresholds ranging from 0.1% to 5% with a resolution of 0.1%. The silhouette method was employed to test (NBclust v.3.0.1), if the known DAN subtype groups were reproduced based on clustering of upstream connectivity. USN clusters were defined to be connected specifically and strongly to a DAN type when the cluster was connected to at least 20% of the neurons constituting that type. A DAN type is, for example, PAM08, which consists of the subtypes PAM08_a through PAM08_e. The connectivity map displaying the input network was generated using Cytoscape v3.9.1. Edge transparency was continuously mapped between the pixel values of 49 and 149 (minimum at 0.1%, maximum at 12.17%). Edge weight was passthrough mapped onto the percentage dendritic input range (0% to 12.17%). Edges were bundled with default Cytoscape parameters (n.o.h. = 3, s.c. = 0.0003, c.t. = 0.3, m.i. = 500).

### Upstream neuron connectivity heat map

For representation on heat maps, the top 200 clusters (450 neurons) providing the strongest dendritic input to all β′2 (PAM02, PAM05 and PAM06), γ4 (PAM08), γ4<γ1γ2 (PAM07), γ5 (PAM01) and γ5β′2a (PAM15) DANs were analysed and clustered by their percentage dendritic input (using base R functions to calculate Manhattan distance metrics and Ward’s clustering criterion). No thresholds were applied. DANs were sorted by their type and percentage dendritic input was normalized by DAN total dendritic input within a row. The graphical representation was generated using the R package ComplexHeatmap v1.10.2.

### Two-photon in vivo calcium imaging

Flies up to 10 days old were briefly anaesthetized for <10 s on ice then mounted with wax onto a custom-made imaging chamber. Each fly was tethered by immobilizing its thorax, legs, and then eyes with wax, whereas its proboscis, maxillary palps, and antennae were left unwaxed. The posterior head capsule was immersed in 1 ml sugar-free HL3-like saline solution^84^ (140 mM NaCl, 2 mM KCl, 4.5 mM MgCl_2_, 1.5 mM CaCl_2_, 5 mM HEPES-NaOH, pH 7.1, 275 mOsm kg^−1^) and opened at room temperature. After surgery, the brain was covered with a 15 μl droplet of sugar-free saline supplemented with 1% agarose and then re-immersed in 1 ml sugar-free saline.

A customized Scientifica Slicescope was equipped with a LUMPLFLN 40× 0.8 NA water-immersion objective (Olympus) and a dichroic beamsplitter (BrightLine, Semrock) with green (500/15 nm, Semrock) and red (578/21 nm, Semrock) filters each followed by GaAsP PMTs (Hamamatsu) to detect GCaMP6f and tdTomato signals respectively. A Ti:sapphire laser (Chameleon Ultra II, Coherent) excited fluorescence using 140 fs pulses centred on 910 nm with an 80 MHz repetition rate. Images of 256 × 256 pixels were acquired at 5.74 Hz under the control of ScanImage 3.8 software^85^ via MATLAB (MathWorks, release 2012a).

### Two-photon imaging stimulus delivery

Odours were delivered on a clean air carrier stream using a custom-designed system^86^, which synchronized odour delivery timing with two-photon image acquisition via LabVIEW (National Instruments). The odours used were MCH and OCT. Each odour (Sigma-Aldrich) was diluted 100× in mineral oil (Sigma-Aldrich) then further blended 1:9 with the clean air stream before being delivered to the tethered fly.

A custom-built feeding stage^24^ delivered a 15 µl to 20 µl droplet towards the freely-moving proboscis of a tethered fly. Each droplet was prepared with 0.4% Brilliant Blue FCF dye (Wako Chemicals) or red amaranth dye (Sigma-Aldrich). Sucrose was delivered at a concentration of 1 M. For Fig. 6f, initial-trial water and post-water sucrose were delivered to each fly with a random order of alternating dye colours. A custom-written LabVIEW subroutine^24^ controlled the feeding stage position by specifying its horizontal and vertical coordinates. Since feeding was voluntary and proboscis position varied between tethered flies, the feeding stage was manually directed by remote control towards the appropriate coordinates at the desired times. Each fly was photographed before surgery and after imaging to confirm that feeding was successful. Flies without visible dye (or without both dyes for flies delivered two stimuli in Fig. 6f) in their digestive tract post-imaging were excluded from the analysis. A Stingray CCD camera (Allied Vision Technologies) recorded fly feeding at 15 Hz during two-photon imaging. These recordings were used to confirm the timing of feeding onset and offset.

### Two-photon imaging analysis

Fiji^81^ was used to demarcate the anatomically distinct β′2, γ4 and γ5 ROIs manually for *R48B04*-driven expression of GCaMP6f and tdTomato. MATLAB (release 2021a) was used for all subsequent processing of the fluorescence signal. For each ROI, the mean intensity of each frame was extracted for each of the two channels (green/GCaMP6f and red/tdTomato) recorded. To control for motion-related artefacts, the mean intensity of each GCaMP6f frame for an ROI was divided by the mean intensity of its corresponding tdTomato frame, yielding *R*, the ratio of the GCaMP6f to tdTomato signal for an ROI.

The resulting ratiometric fluorescence trace *R* for the ROI in each hemisphere was then averaged to yield the mean trace for that ROI in an individual fly. The mean trace was normalized for each stimulus-evoked response by calculating Δ*R/R*_0_, where *R*_0_ is the arithmetic mean of *R* for the 5 s before the onset of each stimulus (10 s before for Fig. 6d) and Δ*R* = *R – R*_0_. For all response curves plotted in Fig. 6b,c,f, the central line is the arithmetic mean of each frame for all samples in a treatment group, and the s.e.m. is displayed as a shaded error bar above and below the central line. The peak of a response to a stimulus was defined to be the maximum value of Δ*R/R*_0_ from 0 s to 20 s after stimulus onset. For all mean difference curves plotted in Fig. 6g, the central line is the difference in the arithmetic means of two treatment groups for each frame, and the 95% confidence interval of the difference is displayed as a shaded error bar above and below the central line.

To compare the effects of physiological state manipulations on baseline calcium signals in Extended Data Fig. 10, only the initial-trial water and initial-trial sucrose samples from Fig. 6f were analysed. The baseline calcium signal was defined to be the arithmetic mean of the GCaMP6f fluorescence for the 60 s before stimulus onset. The mean signal for each ROI in each hemisphere was calculated over this time frame, as was that of an additional ‘background’ ROI in each hemisphere for a non-implicated region. The mean signal for each ROI was then divided by the mean signal of the background ROI in the corresponding hemisphere. The mean ROI signal (relative to its corresponding background ROI) was then averaged across both hemispheres to yield the mean baseline signal for that ROI in each fly.

### Statistics

Statistical analyses were performed in MATLAB (release 2021a) or GraphPad Prism 8.4.3. All behavioural and confocal data were analysed with a two-sided unpaired t-test, a one-way ANOVA followed by multiple comparisons (Tukey’s HSD), or a two-way ANOVA followed by multiple comparisons with Šidák’s correction or Tukey’s HSD. Homoscedasticity among genotypes was not assumed for the multiple two-sided unpaired t-tests performed in Extended Data Fig. 4a with Holm–Šidák’s correction. For two-photon imaging data, peak responses in Fig. 6d were analysed with a two-way repeated measures ANOVA for odours (in which both regions and odours were matched for individual flies) or a one-way repeated measures ANOVA for sucrose (in which regions were matched for individual flies), followed by Tukey’s HSD. For the mean differences in Fig. 6g, frames for which the 95% confidence interval of the difference did not include zero (that is, a two-sided unpaired *t*-test) were considered statistically significant without correcting for multiple comparisons and denoted with a plus symbol (+) for each corresponding frame. Baseline signals in Extended Data Fig. 10 were analysed with a two-way repeated measures ANOVA (in which regions were matched for individual flies) followed by Tukey’s HSD. Sphericity was assumed for all matching observations from imaging data analysed with repeated measures ANOVA. All analyses of variance, mean difference, and descriptive statistics are described in Supplementary Tables 3–5.

### Reporting summary

Further information on research design is available in the Nature Portfolio Reporting Summary linked to this article.

## Online content

Any methods, additional references, Nature Portfolio reporting summaries, source data, extended data, supplementary information, acknowledgements, peer review information; details of author contributions and competing interests; and statements of data and code availability are available at 10.1038/s41586-023-06671-8.

### Supplementary information

Supplementary Information A full guide to Supplementary Tables 1–6 and Supplementary Video 1 (tables and video supplied separately).

Reporting Summary

Supplementary Table 1 All 1,996 USNs to β′2, γ4 and γ5 PAM DANs, arranged by combined connectivity strength in descending order.

Supplementary Table 2 All 1,718 USNs to only β′2 and γ4 PAM DANs, arranged by combined connectivity strength in descending order.

Supplementary Table 3 Analyses of variance (ANOVA) for all figures and extended data figures.

Supplementary Table 4 Comparisons of differences between the means of specific groups for all figures and extended data figures.

Supplementary Table 5 Descriptive statistics and individual values for all behavioural results, confocal cell counts, peak calcium responses, and mean baseline calcium signals in figures and extended data figures.

Supplementary Table 6 Individual calcium responses for all imaging data in Fig. 6.

Supplementary Video 1 Volumetric reconstructions of β′2 and γ4 DANs and all their USNs.

## Data Availability

Supplementary Table 5 contains all individual data values plotted in the figures and extended data figures, whereas Supplementary Table 6 contains all individual calcium imaging responses for the imaging data in Fig. 6. The transcriptome dataset used for Fig. 1 has been deposited in BioProject under accession code PRJNA1008630. The connectome dataset for the *Drosophila* hemibrain (v1.2.1) used for Fig. 5 and Extended Data Figs. 7 and 8 is publicly available at https://neuprint.janelia.org. Supplementary Tables 1 and 2 contain all USNs identified and their individual percentage dendritic inputs to each DAN.

## Code availability

All custom MATLAB code used to analyse the behavioural and calcium imaging data is available on request from K.D.J. The code used to analyse the transcriptome data is available at https://satijalab.org/seurat/, and the code used to analyse the connectome data is available at https://natverse.org/ and https://pypi.org/project/navis/.
