# Publisher Correction: Dopaminergic systems create reward seeking despite adverse consequences

**DOI:** 10.1038/s41586-023-06876-x

**Published:** 2023-11-27

**Authors:** Kristijan D. Jovanoski, Lucille Duquenoy, Jessica Mitchell, Ishaan Kapoor, Christoph D. Treiber, Vincent Croset, Georgia Dempsey, Sai Parepalli, Paola Cognigni, Nils Otto, Johannes Felsenberg, Scott Waddell

**Affiliations:** 1 Centre for Neural Circuits and Behaviour, University of Oxford, Oxford, UK; 2 Present Address: Friedrich Miescher Institute for Biomedical Research, Basel, Switzerland; 3 Present Address: Department of Biosciences, Durham University, Durham, UK; 4 Present Address: Northern Medical Physics and Clinical Engineering, Newcastle upon Tyne Hospitals NHS Trust, Newcastle upon Tyne, UK; 5 Present Address: Institute of Anatomy and Molecular Neurobiology, Westfälische Wilhelms-University, Münster, Germany

**Keywords:** Motivation, Reward

Correction to: *Nature* 10.1038/s41586-023-06671-8 Published online 25 October 2023

In the version of the article initially published, the present address for Johannes Felsenberg was incorrect, and has now been updated to Friedrich Miescher Institute for Biomedical Research, Basel, Switzerland in the HTML and PDF versions of the article.

