# Supplementary material for: Dopaminergic systems create reward seeking despite adverse consequences
Source: Nature. 2023 Oct 25;623(7986):356–65. doi: 10.1038/s41586-023-06671-8 (PMC10632144; doi:10.1038/s41586-023-06671-8)
Supplement: Supplementary file 1 — A full guide to Supplementary Tables 1–6 and Supplementary Video 1 (tables and video supplied separately). [file 41586_2023_6671_MOESM1_ESM.pdf]

nature portfolio

https://doi.org/10.1038/s41586-023-06671-8

Supplementary information

Dopaminergic systems create reward seeking despite adverse consequences

In the format provided by the authors and unedited

Dopaminergic systems create reward seeking despite adverse consequences

Kristijan D. Jovanoski[1✉], Lucille Duquenoy[1], Jessica Mitchell[1,2], Ishaan Kapoor[1], Christoph D. Treiber[1], Vincent Croset[1,3], Georgia Dempsey[1], Sai Parepalli[1], Paola Cognigni[1,4], Nils Otto[1,5], Johannes Felsenberg[1,2], Scott Waddell[1✉]

[1] Centre for Neural Circuits and Behaviour, University of Oxford, Oxford, OX1 3SR, UK.
[2] Present address: Friedrich Miescher Institute for Biomedical Research, Basel, 4058 Switzerland.
[3] Present address: Department of Biosciences, Durham University, Durham, DH1 3LE, UK.
[4] Present address: Northern Medical Physics and Clinical Engineering, Newcastle upon Tyne Hospitals NHS Trust, Newcastle upon Tyne, NE7 7DN, UK.
[5] Present address: Institute of Anatomy and Molecular Neurobiology, Westfälische Wilhelms-University, 48149 Münster, Germany.
✉ e-mail: kdjovanoski@gmail.com; scott.waddell@cncb.ox.ac.uk

Supplementary Information Guide

Supplementary Video 1 | Volumetric reconstructions of β′2 and γ4 DANs and all their upstream neurons. Volumetric reconstructions of β′2 and γ4 PAM DANs (in blue and red respectively) are displayed, followed by their orientation within the mushroom body neuropil (grey) and then within the entire FlyEM hemibrain neuropil (light grey). Subsequently, all 1718 upstream neurons (USNs) to β′2 and γ4 PAM DANs are displayed, initially within the hemibrain neuropil for orientation. The 402 USNs comprising the top 200 clusters most strongly connected to β′2 and γ4 PAM DANs are coloured by neurite location (matching those in Fig. 5b & c and Extended Data Fig. 7), while the remaining USNs are displayed in grey. Finally, the top 200 USN clusters are visualized in a 360-degree rotation around the dorsoventral axis.

Supplementary Table 1 | All 1996 upstream neurons to β′2, γ4, and γ5 PAM DANs, arranged by combined connectivity strength in descending order. Values represent the percentage dendritic input (i.e., connectivity strength as a percentage of the total dendritic input of a DAN) from individual USN clusters (rows) to individual DANs (columns). Clusters are named according to and compared with Neuprint v1.2.1 neuron types; MDBL axons were clustered separately (see Methods for details). The first 450 rows of the table

correspond to the USNs that constitute the top 200 clusters with the strongest dendritic input to β′2, γ4, and γ5 PAM DANs (used for Fig. 5c & Extended Data Fig. 8).

Supplementary Table 2 | All 1718 upstream neurons to only β′2 and γ4 PAM DANs, arranged by combined connectivity strength in descending order. Values and clusters are represented as per Supplementary Table 1. The first 402 rows of the table correspond to the USNs that constitute the top 200 clusters with the strongest dendritic input to only β′2 and γ4 PAM DANs (used for Fig. 5b & Extended Data Fig. 7).

Supplementary Table 3 | Analyses of variance (ANOVA) for all Figures and Extended Data Figures. Group numbers correspond to their appearance from left to right in each figure panel. Sphericity was assumed for all matched observations analysed with repeated measures ANOVA (rANOVA). Only the interaction result is presented for analyses in which the interaction between treatment and the other factor (genotype, test session, or time before test) was found to be statistically significant.

Supplementary Table 4 | Comparisons of differences between the means of specific groups for all Figures and Extended Data Figures. Group A and B numbers and short names correspond to the appearance of specific groups from left to right in each figure panel. Note that some comparisons involve multiple specific groups (linked with ampersands) pooled together to form Groups A and B. Statistical tests are named with corrections for multiple comparisons in parentheses where applicable. The difference between means (Mean Diff.) was calculated as (Mean A – Mean B) with corresponding 95% confidence intervals (Lower CI and Upper CI). The standard error of the difference between means (SE Diff.) is also provided, as are the sample sizes (n) for each group and degrees of freedom (df). The test value corresponds to the *q*-value for each Tukey's HSD test or the *t*-value for all other tests, whereas the *p*-value provided for each test is the value adjusted after multiple comparisons where applicable.

Supplementary Table 5 | Descriptive statistics and individual values for all behavioural results, confocal cell counts, peak calcium responses, and mean baseline calcium signals in Figures and Extended Data Figures. Individual groups (rows) are described with protocol summaries, descriptive statistics, and source data (columns). Group numbers, short names, and genotypes correspond to the appearance of specific groups from left to right in each figure panel and match those used in Supplementary Table 4. All behavioural data groups have summaries of their physiological state, training protocol, test duration (in seconds), and each choice during testing. All calcium imaging groups have

summaries of their physiological state, imaging region of interest, and presentation stimulus. Descriptive statistics are the arithmetic mean, standard deviation, standard error of the mean, 95% confidence interval of the mean (Lower CI and Upper CI), the minimum and maximum values, and the sample size (*n*). Numbered columns to the right of the sample size column correspond to the data values of individual samples, and matched observations (for calcium imaging data) where applicable.

Supplementary Table 6 | Individual calcium responses for all imaging data in Fig. 6. Individual calcium responses (rows) are described by genotype, physiological state, unique sample identifier, imaging region of interest, presentation stimulus, number of frames, and source data (columns). Rows with the same sample identifier correspond to recordings from the same fly. Numbered columns to the right of the number of frames column correspond to the $\Delta R / R_0$ values of individual frames, and simultaneous observations (over three rows) of β′2, γ4, and γ5n DAN responses to specific odours or droplets presented to a particular fly (stimulus presentation protocols described in Fig. 6).
