# Supplementary material for: Dopaminergic systems create reward seeking despite adverse consequences
Source: Nature. 2023 Oct 25;623(7986):356–65. doi: 10.1038/s41586-023-06671-8 (PMC10632144; doi:10.1038/s41586-023-06671-8)
Supplement: Supplementary file 2 — Reporting Summary [file 41586_2023_6671_MOESM2_ESM.pdf]

nature portfolio

Corresponding author(s): Kristijan Jovanoski & Scott Waddell

Last updated by author(s): Aug 23, 2023

Reporting Summary

Nature Portfolio wishes to improve the reproducibility of the work that we publish. This form provides structure for consistency and transparency in reporting. For further information on Nature Portfolio policies, see our Editorial Policies and the Editorial Policy Checklist.

Statistics

For all statistical analyses, confirm that the following items are present in the figure legend, table legend, main text, or Methods section.

n/a	Confirmed	
☐	☒	The exact sample size (*n*) for each experimental group/condition, given as a discrete number and unit of measurement
☐	☒	A statement on whether measurements were taken from distinct samples or whether the same sample was measured repeatedly
☐	☒	The statistical test(s) used AND whether they are one- or two-sided<br>*Only common tests should be described solely by name; describe more complex techniques in the Methods section.*
☒	☐	A description of all covariates tested
☐	☒	A description of any assumptions or corrections, such as tests of normality and adjustment for multiple comparisons
☐	☒	A full description of the statistical parameters including central tendency (e.g. means) or other basic estimates (e.g. regression coefficient) AND variation (e.g. standard deviation) or associated estimates of uncertainty (e.g. confidence intervals)
☐	☒	For null hypothesis testing, the test statistic (e.g. *F*, *t*, *r*) with confidence intervals, effect sizes, degrees of freedom and *P* value noted<br>*Give P values as exact values whenever suitable.*
☒	☐	For Bayesian analysis, information on the choice of priors and Markov chain Monte Carlo settings
☒	☐	For hierarchical and complex designs, identification of the appropriate level for tests and full reporting of outcomes
☒	☐	Estimates of effect sizes (e.g. Cohen's *d*, Pearson's *r*), indicating how they were calculated

Our web collection on statistics for biologists contains articles on many of the points above.

Software and code

Policy information about availability of computer code

Data collection	Two-photon imaging data were collected using LabVIEW and ScanImage 3.8 under the control of MATLAB (release 2012a). Confocal imaging data were collected using the Leica LAS AF suite. Connectomics data were collected from the hemibrain nanoscale connectome EM dataset (v1.2.1, neuprint.janelia.org) using the NAVis Python package.
Data analysis	Behavioural and two-photon imaging data were analysed using customized scripts written in MATLAB (release 2021a) or using GraphPad Prism 8.4.3. The customized MATLAB scripts supporting the manuscript are available from K.D.J. on request. Single-cell RNA sequencing data were clustered using the Seurat v3 R package. Confocal imaging data were analysed using Fiji. Connectomics data were analysed/visualized using the R-based natverse package nat.flybrains v.1.7.4, the R package ComplexHeatmap v1.10.2, the Python-based NAVis package v1.3.0, Blender v3.2.2, and Cytoscape v3.9.1.

For manuscripts utilizing custom algorithms or software that are central to the research but not yet described in published literature, software must be made available to editors and reviewers. We strongly encourage code deposition in a community repository (e.g. GitHub). See the Nature Portfolio guidelines for submitting code & software for further information.

Data

Policy information about availability of data

All manuscripts must include a data availability statement. This statement should provide the following information, where applicable:

- Accession codes, unique identifiers, or web links for publicly available datasets
- A description of any restrictions on data availability
- For clinical datasets or third party data, please ensure that the statement adheres to our policy

All individual data values plotted and all individual calcium imaging responses are presented in Supplementary Tables 5 and 6. The transcriptome dataset has been deposited in BioProject under accession code PRJNA1008630. The connectome dataset for the Drosophila hemibrain (v1.2.1) is publicly available at https://neuprint.janelia.org. Supplementary Tables 1 & 2 contain all upstream neurons identified and their individual percentage dendritic inputs to each DAN.

Research involving human participants, their data, or biological material

Policy information about studies with human participants or human data. See also policy information about sex, gender (identity/presentation), and sexual orientation and race, ethnicity and racism.

Reporting on sex and gender	N/A
Reporting on race, ethnicity, or other socially relevant groupings	N/A
Population characteristics	N/A
Recruitment	N/A
Ethics oversight	N/A

Note that full information on the approval of the study protocol must also be provided in the manuscript.

Field-specific reporting

Please select the one below that is the best fit for your research. If you are not sure, read the appropriate sections before making your selection.

☒ Life sciences ☐ Behavioural & social sciences ☐ Ecological, evolutionary & environmental sciences

For a reference copy of the document with all sections, see nature.com/documents/nr-reporting-summary-flat.pdf

Life sciences study design

All studies must disclose on these points even when the disclosure is negative.

Sample size	Sample sizes are stated in each figure legend and Supplementary Table 5. No statistical methods were used to predetermine sample sizes but our sample sizes are similar to or greater than those reported in previous publications in the field (e.g. Lin et al., 2014; Huetteroth et al., 2015; Felsenberg et al., 2017).
Data exclusions	No data were excluded from behavioural or imaging experiments, with the exception of flies that did not feed during two-photon imaging (based on pre-established exclusion criteria described in Methods: Two-photon imaging stimulus delivery).
Replication	Experiments were replicated at least twice independently and all attempts at replication were successful.
Randomization	Groups of flies were tested in parallel and in a randomized order.
Blinding	Data collection and analyses were not performed blind to the conditions of the experiments due to the unambiguous nature of some measurements or because the experimenters required this information to carry out the correct protocols.

Reporting for specific materials, systems and methods

We require information from authors about some types of materials, experimental systems and methods used in many studies. Here, indicate whether each material, system or method listed is relevant to your study. If you are not sure if a list item applies to your research, read the appropriate section before selecting a response.

Materials & experimental systems

n/a	Involved in the study
☒	☐ Antibodies
☒	☐ Eukaryotic cell lines
☒	☐ Palaeontology and archaeology
☐	☒ Animals and other organisms
☒	☐ Clinical data
☒	☐ Dual use research of concern
☒	☐ Plants

Methods

n/a	Involved in the study
☒	☐ ChIP-seq
☒	☐ Flow cytometry
☒	☐ MRI-based neuroimaging

Animals and other research organisms

Policy information about studies involving animals; ARRIVE guidelines recommended for reporting animal research, and Sex and Gender in Research

Laboratory animals	Fruit flies (Drosophila melanogaster) up to 12 days old were used in the study.
Wild animals	The study did not involve wild animals.
Reporting on sex	Mixed-sex populations were used for all behavioural experiments. Flies were matched for sex in all groups for two-photon imaging experiments. The connectome dataset is derived from the hemibrain of a single female fly.
Field-collected samples	The study did not involve samples collected from the field.
Ethics oversight	No ethical approval or guidance was required.

Note that full information on the approval of the study protocol must also be provided in the manuscript.
